# Transcriptome and metabolome analyses revealed the main profiles contributing the mild aroma characteristics of *Artemisia stolonifera*

**DOI:** 10.3389/fpls.2025.1713657

**Published:** 2025-11-26

**Authors:** Ye Cao, Yan Ren, Ye Wang, Hui Li

**Affiliations:** 1 Jiangxi Key Laboratory for Sustainable Utilization of Chinese Materia Medica Resources, Institute of Traditional Chinese Medicine Health Industry, China Academy of Chinese Medical Sciences, Nanchang, China; 2 Jiangxi Institute of Traditional Chinese Medicine Health Industry, Nanchang, China; 3 Institute of Chinese Materia Medica, China Academy of Chinese Medical Sciences, Beijing, China

**Keywords:** *Artemisia stolonifera*, *Artemisia argyi*, metabolome, transcriptome, terpenoid biosynthesis, flavonoid biosynthesis

## Abstract

*Artemisia stolonifera*, identified as an original source of mugwort leaf during the fourth national medicinal resource investigation in China, remains considerably understudied compared to the well-characterized *Artemisia argyi*, despite its distinctive mild aroma and potential therapeutic value. The lack of systematic comparative analyses on their active compounds and underlying biosynthetic mechanisms has limited the application and development of *A. stolonifera*. To address this gap, we conducted integrated metabolomic and transcriptomic analyses of leaves from both species. Using GC-MS for targeted profiling of volatile organic compounds and UPLC-ESI (-Q TRA)-MS/MS for flavonoids and lignin pathway intermediates, we identified 1,728 differentially accumulated metabolites (DAMs). Transcriptome sequencing generated 37.61 Gb of clean data, revealing 18,000 differentially expressed genes (DEGs). Kyoto Encyclopedia of Genes and Genomes analysis demonstrated significant divergence in terpenoid and flavonoid biosynthesis pathways between the two species. *A. stolonifera* exhibited accumulation sesquiterpenoids, consistent with the concerted upregulation of mevalonate pathway genes (e.g., *AACT1-4*, *HMGR1-3*) and sesquiterpene synthases (*BAS1*, *LUP2*, *CAMS1*, *XF1*). Conversely, *A. argyi* exhibited enrichment of monoterpenoids and flavonoids, associated with elevated expression of methylerythritol phosphate pathway genes (*DXS2*, *DXR1-5*), monoterpenoid biosynthesis genes (*SDR2-4*, *TPS14*), and flavonoid biosynthesis genes (e.g., *CHS1-2*, *CHI*, *F3H1-3*). This study elucidates the divergence of genetic and metabolic basis governing bioactive compound biosynthesis between these species, revealing that the characteristically mild aroma of *A. stolonifera* results from its lower volatile oil content and reduced levels of intense monoterpenoids. These insights provide a critical foundation for evaluating the medicinal value and supporting the safe utilization of *A. stolonifera*.

## 1 Introduction

Moxibustion, a cornerstone therapeutic modality in traditional Chinese medicine (TCM) (Tian et al., 2020), utilizes moxa wool derived primarily from the dried leaves of *Artemisia argyi* (designated “mugwort leaf” in the Chinese Pharmacopoeia) as its combustion. During the fourth national medicinal resource investigation in China, our research team identified *Artemisia stolonifera* as an original plant source for mugwort leaf due to its superior combustion properties. Multidisciplinary analyses, including textual examination of classical pharmacopeias, geographical provenance assessment, and field investigations, confirmed that *A. stolonifera* corresponds to the historical Jiu-Niu-Cao documented in classical pharmacopeia records (*Ben Cao Tu Jing*, 1061 CE) (Luo et al., 2020b). However, clear botanical descriptions distinguishing *A. stolonifera* from *A. argyi* remain relatively scarce, impeding accurate species identification and quality control. Importantly, while both *A. argyi* and *A. stolonifera* have been historically used for moxibustion and medicine, only *A. argyi* is currently recorded in the Chinese Pharmacopoeia for the medicinal use of Artemisiae Argyi Folium and has the largest cultivation scale. This is largely due to insufficient comparative data on their chemical composition profiles and associated bioactivities.

Chemical investigations have demonstrated that the bioactive constituents in *Artemisia* species predominantly consist of terpenoids, flavonoids, polysaccharides, coumarins, sesquiterpene lactones, and organic acids (Abad et al., 2012; Anwar et al., 2016; Han et al., 2017; Zhang et al., 2018; Jiang et al., 2019). These secondary metabolites exhibit a wide range of pharmacological properties, including antioxidant, antitumor, anti-inflammatory, anticoagulant, anti-osteoporotic, and immunomodulatory activities (Kim et al., 2015b, 2015a; Ge et al., 2016; Yun et al., 2016; Lv et al., 2018). Consequently, the medicinal efficacy of these plants correlates directly with their metabolite profiles. However, systematic comparative investigations of the specialized secondary metabolites, particularly volatile terpenoids and non-volatile bioactive components such as flavonoids, between *A. stolonifera* and *A. argyi* remain notably limited. Although *A. stolonifera* has documented therapeutic uses, its application remains restricted compared to *A. argyi*, primarily due to the absence of comprehensive comparative metabolomic studies. Current research on *A. stolonifera* has predominantly focused on characterizing its volatile components using techniques such as HS-SPME-GC-MS, revealing significant differences when compared to *A. argyi* (Li et al., 2022, 2023). Notably, *A. stolonifera* is distinguished by a mild and fresh aroma, a characteristic attributed to its lower volatile oil content. However, despite the recognized importance of terpenoids and flavonoids in the therapeutic efficacy of *A. stolonifera*, comprehensive metabolomic profiling targeting these key constituents is notably lacking. This deficiency was the primary reason terpenoids and flavonoids were utilized as comparative indices in this investigation. Previous studies on *A. argyi* have often prioritized the volatile secondary metabolite profiling of its various tissues; although fewer in number, studies examining the biosynthetic pathways have established a foundational understanding, particularly concerning the main flavonoid biosynthetic pathway, which is known to critically influence the quality of *A. argyi*. In contrast, the biosynthetic pathways and regulatory networks governing the production of these critical metabolites in *A. stolonifera*, especially in comparison to *A. argyi*, remain poorly elucidated. This lack of understanding regarding the metabolic machinery of *A. stolonifera* hinders a comprehensive assessment of its full potential for medicinal or moxibustion applications.

Headspace solid-phase microextraction coupled with gas chromatography-mass spectrometry (HS-SPME-GC-MS) provides exceptional sensitivity in characterizing volatile terpenoids in *A. stolonifera*. However, its inability to detect non-volatile metabolites necessitates the use of complementary analytical platforms. To address the limitations of GC-based methods for non-volatile metabolites, this study employs ultra-performance liquid chromatography coupled with mass spectrometry (UPLC-MS). Specifically, UPLC-ESI-MS facilitates comprehensive quantification of polar bioactive compounds, while UPLC-ESI-QTRAP-MS enhances the precision of targeted biomarker quantification through multiple reaction monitoring (MRM). This methodological integration, validated by Luo et al. (2020a), who identified chlorogenic acids and flavonoids as dominant non-volatiles, underscores the necessity of employing UPLC techniques to construct comprehensive flavonoid metabolomes for comparative structural characterization. This integrated metabolomic platform is vital for developing a detailed metabolome, particularly for flavonoids and other essential non-volatile phytochemicals, thereby enabling high-resolution structural characterization crucial for establishing bioactivity-compound relationships and quality control protocols for *A. stolonifera*. When integrated with transcriptomics, a multi-omics strategy offers systems-level insights into the regulatory mechanisms that govern metabolite accumulation and gene expression. Such multi-omics integration can effectively reveal the chemical basis of pharmacological properties and species-specific metabolic patterns (Chen et al., 2021; Du et al., 2021; Zhang et al., 2022).

In summary, this study employs integrated transcriptomic and metabolomic analyses of functional leaves to systematically investigate the secondary metabolism of *A. stolonifera* in comparison to *A. argyi*. We hypothesized that the distinct mild aroma and metabolic profile of *A. stolonifera*, compared to *A. argyi*, are primarily governed by species-specific differences in the expression of key genes involved in the terpenoid and flavonoid biosynthesis pathways, leading to significantly different abundances of volatile compounds. To validate this hypothesis, our study comprehensively characterizes and compares the metabolite profiles of volatile terpenoids and non-volatile key metabolites between the two species. We aim to delineate species-specific transcriptional regulation patterns, with particular focus on genes involved the terpenoid backbone biosynthesis pathways and the flavonoid biosynthetic pathway. Furthermore, we will construct integrated gene-metabolite correlation networks to elucidate the molecular mechanisms responsible for the observed metabolic divergence between *A. stolonifera* and *A. argyi*. These findings will significantly advance our understanding of the genetic and metabolic basis governing the biosynthesis of bioactive compounds in economically and medicinally significant *Artemisia* species, providing fundamental insights for evaluating the potential and safe utilization of *A. stolonifera*.

## 2 Materials and methods

### 2.1 Plant materials

Fresh leaves of *A. stolonifera* (AS) and *A. argyi* (AA) were collected for this study, identified by Academician Luqi Huang from the China Academy of Chinese Medical Sciences. Both species were initially cultivated in February 2020 at the Standardized Planting Base of Jiu-Niu-Cao in Sunjia Village, Zhangshu City, Jiangxi Province. After 20 days of growth, the plants were transplanted to Shennongling Herb Science and Technology Park (28.94°N, 115.81°E) in Nanchang City, Jiangxi Province, for field acclimatization and domestication. The plants were grown under standardized cultivation conditions for over 45 days prior to sampling in October 2024. The samples were immediately placed in 15 mL centrifuge tubes, flash-frozen in liquid nitrogen, and stored at -80°C until analysis. Three biological replicates per species were processed.

### 2.2 RNA-sequencing based on transcriptomic detection

Total RNA was extracted from 100 mg of leaf tissue using the RNAprep Pure Plant Plus Kit (TIANGEN, Beijing, China), following the manufacturer’s instructions. The integrity of the RNA was verified through 1.5% agarose gel electrophoresis and assessed using a NanoDrop 2000 (OD260/280 ≥ 1.8). Libraries were constructed from 3 μg of total RNA per sample and sequenced on the Illumina NovaSeq 6000.

Six RNA libraries (AS-1, AS-2, AS-3, AA-1, AA-2, and AA-3) were constructed and analyzed. The HISAT2 v2.0.5 software (Mortazavi et al., 2008) was utilized to map reads to the reference genome of *A. argyi*. The assembly of new transcripts was performed using StringTie v1.3.0 (Pertea et al., 2015). Gene expression was quantified as fragments per kilobase of transcript per million mapped reads (FPKM) (Bray et al., 2016) using featureCounts v1.5.0-p3 (Liao et al., 2014; Love et al., 2014). Differentially expressed genes (DEGs) were identified using DESeq2 v1.20.0 (Anders and Huber, 2010) (|log_2_FC| ≥ 1 and a false discovery rate (FDR) < 0.05). Functional annotation was conducted using the Gene Ontology (GO), Kyoto Encyclopedia of Genes and Genomes (KEGG), and Pfam databases. Enrichment analysis was performed with clusterProfiler v3.8.1 (*p* < 0.05). Heatmaps were generated by TBtools-II (Chen et al., 2023).

### 2.3 Volatile components detection using HS-SPME-GC-MS metabolomic method

Volatile organic compounds (VOCs) were extracted from 500 mg of leaf powder using 20 mL of head-space l in a SPME process with a 120 μm DVB/CAR/PDMS fiber (Supelco). During the SPME analysis, each vial was maintained at 60°C for 5 min, after which the fiber was exposed to the headspace at the same temperature for 15 minutes. GC-MS (Agilent Technologies Inc., CA, USA) analysis was conducted using an Agilent 8890/7000D system equipped with a DB-5MS column (30 m × 0.25 mm × 0.25 μm). Helium (purity > 99.999%) was used as the carrier gas flowing at a constant rate of 1.2 mL·min^-1^. The injector temperature was set to 250°C. The temperature program was as follows: the column thermostat was held at 40°C for 3.5 min, then increased to 100°C at a rate of 10°C·min^-1^, further increased to 180°C at 7°C·min^-1^, escalated to 280°C at 25°C min^-1^, and held at this final temperature for 5 min. Mass spectra were recorded in electron impact (EI) ionization mode at 70 eV. The temperatures of the MS electron impact ion source and quadrupole were set to 230 and 150°C, respectively. Mass data were collected in ion monitoring (SIM) mode for the identification and quantification of analytes, with a solvent delay time of 5 min.

The raw data were obtained following the automatic and manual integration of the peak area using MassHunter Quantitative Analysis vB.07.00 (Yuan et al., 2022). Metabolites were identified by comparing mass spectra with the NIST20 library (similarity > 80%) and linear retention indices (RI) from the data system libraries.

### 2.4 Non-volatile components using UPLC-ESI (-Q TRA)-MS/MS metabolomic method

Fresh leaves of *A. stolonifera* and *A. argyi* were lyophilized using a vacuum freeze-dryer. The dried leaves were ground into a powder (30 Hz, 1.5 min) using a grinder. For UPLC-ESI-MS/MS analysis, 50 mg of the powder was extracted with 1.2 mL of 70% aqueous methanol. Following centrifugation at 12,000 rpm for 3 min, the supernatant was aspirated with a syringe and filtered through a 0.22 μm organic-phase membrane. For UPLC-ESI-Q TRAP-MS/MS analysis, 100 mg of the powder was extracted with 0.6 mL of 70% aqueous methanol at 4 °C for 12 h. After centrifugation (10, 000 rpm for 10 min), the supernatant was aspirated and filtered.

Chromatographic separation was achieved using Agilent SB-C18 columns (100 × 2.1 mm, 1.8 μm) on two platforms: (1) UPLC system (UPLC, ExionLC™ AD) coupled with a tandem mass spectrometry system (Sciex QTRAP 6500+), and (2) UPLC system (UPLC, Shim-pack UFLC SHIMADZU CBM30A system) with a tandem mass spectrometry system (Applied Biosystems 4500 Q TRAP). Both systems employed identical mobile phases: solvent A (pure water with 0.1% formic acid) and solvent B (acetonitrile with 0.1% formic acid), with a flow rate of 0.35 mL·min^-1^ and a column temperature maintained at 40 °C. The analytical conditions were as follows: gradient program starting at 5% B at 0 min, increasing to 95% B at 9 min, maintaining 95% B for 1 min, then adjusting to 5.0% B within 1.1 min and maintaining it for 2.9 min.

For QQQ-MS/MS analysis (injection volume 2 μL), the ESI source conditions were as follows: ion source temperature at 500°C; ion spray voltage ranging from 5,500 to 4,500 V; ion source gas I, gas II, and curtain gas set at 50, 60, and 25.0 psi, respectively, with medium collision gas (N_2_) setting. For QTRAP-MS/MS analysis (4 μL injection), the ESI source conditions were as follows: ion source temperature at 550°C; ion spray voltage ranging from 5500 to 4500 V; ion source gas I, gas II, and curtain gas set at 50, 60, and 30.0 psi, respectively, with a collision gas setting of 5 psi. Both platforms acquired data in MRM mode. The qualitative and quantitative analyses of metabolites integrated both public databases and a self-built database of metabolite information.

The GC-/UPLC-/LC-MS data were imported into Microsoft Excel 2019 for data organization, processing, and statistical analysis. Multivariate analyses including principal component analysis (PCA) and orthogonal partial least squares discriminant analysis (OPLS-DA), were performed using R packages. Differentially accumulated metabolites (DAMs) were identified based on the thresholds of |log_2_(FC)| ≥ 1, FDR < 0.05, and variable importance in projection (VIP) ≥ 1.

### 2.5 Combined correlation analysis of DAMs and DEGs

Hierarchical clustering and KEGG pathway enrichment analyses were conducted using clusterProfiler (v4.0.5). All DEGs and DAMs that were associated with KEGG pathways were utilized. The Pearson correlation coefficients between DAMs and DEGs were calculated using the Psych package in R software. Significant pairs (r > 0.9, p < 0.05) were subsequently converted to mutual ranks using scripts available on GitHub (jwisecav/coexp-pipe).

### 2.6 Quantitative real-time polymerase chain reaction

Total RNA was extracted from 100 mg of frozen leaf tissue using the RNAprep Pure Plant Plus Kit (TIANGEN, Beijing, China) according to the manufacturer’s specifications, with integrity and concentration verified using a NanoDrop 2000 (Thermo Fisher Scientific). First-strand cDNA was synthesized from 1 μg of RNA with the PrimeScript™ RT Kit with gDNA Eraser (Perfect Real Time) (Takara Bio Inc., Shiga, Japan) under optimized conditions: 42°C for 2 min, 37°C for 15 min, and 85°C for 5 s. Amplification was performed on a CFX96 Real-Time PCR Detection System (Bio-Rad Laboratories, Hercules, CA) and included initial denaturation at 95°C for 30 s, followed by 40 cycles of 95°C for 5 s and 60°C for 30 s, concluding with melt curve analysis from 65°C to 95°C at a rate of 0.5°C·s^-1^, using TB Green^®^ Premix Ex Taq™ II (Tli RNaseH Plus) (Takara Bio Inc., Shiga, Japan). The qPCR reactions (25 μL) contained 12.5 μL of TB Green Master Mix, 20 μM gene-specific primers (**Supplementary Table S1**, synthesized by Sangon Biotech, Shanghai, China), and 2 μL of diluted cDNA template. The constitutively expressed *AaActin* served as the internal control for Ct value normalization. Relative quantification was determined using the 2^-ΔΔCT^ method with efficiency correction, employing three biological and technical replicates.

## 3 Results

### 3.1 Botanical description of two similar species between *A. stolonifera* and *A. argyi*

**Figure 1:**
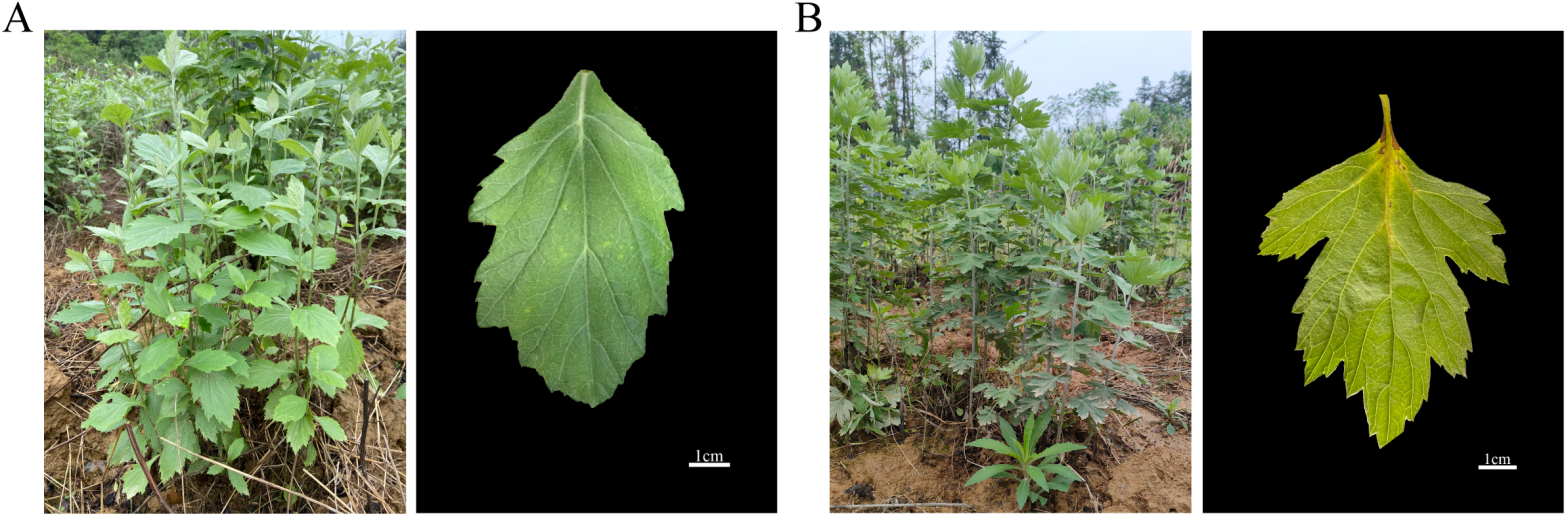
Morphological characterization and leaf of *A. stolonifera***(A)** and *A. argyi****(*B*)***.

*A. stolonifera* and *A. argyi* are perennial herbs belonging to the genus *Artemisia*, exhibiting distinct morphological characteristics. *A. stolonifera* typically reaches a height of 50–120 cm, characterized by sparse gray arachnoid pubescence or a glabrescent appearance. The middle stem leaves are nearly sessile and can be described as obovate-elliptic, ovate-elliptic, or ovate, often exhibiting 2- or 3-cleft or -partite structures with coarse teeth, and are gland-dotted on the adaxial surface (**Figure 1A**). In contrast, *A. argyi* is taller, ranging from 80–250 cm, and features numerous lateral roots with short apical branching, also covered in gray arachnoid pubescence. Its middle stem leaves are ovate, triangular-ovate, or subrhombic, with the adaxial surfaces are incanous pubescent and white gland-dotted. These leaves are generally 1- or 2-pinnatipartite or -cleft (**Figure 1B**). Furthermore, we observed that *A. stolonifera* emits a mild and fresh aroma, which is distinctly different from the intense odor of *A. argyi*. To systematically investigate these phytochemical differences, we analyzed both the volatile and non-volatile components of each species. The volatile profiling aimed to elucidate the specific compositional basis for the characteristically mild aroma of *A. stolonifera*, while flavonoid quantification assessed its potential for non-volatile bioactive constituents. This combined approach provides a comprehensive chemical foundation for understanding the traditional and potential applications of both species, highlighting how the unique sensory characteristics of *A. stolonifera* arise from its distinctive volatile metabolome.

### 3.2 Differential expression of RNA sequencing between *A. stolonifera* and *A. argyi*

To elucidate the molecular mechanisms underlying the differences in secondary metabolism between *A. stolonifera* and *A. argyi*, we conducted comparative transcriptome analysis. A total of 38.25 Gb of raw bases were obtained (**Supplementary Table S2**). After quality filtering, 37.61 Gb of clean data were acquired, with individual sample data ranging from 5.87 to 6.75 Gb. High sequencing quality was confirmed, with Q30 scores exceeding 96.3% and an average GC content of 43.36% (**Supplementary Table S2**). *De novo* assembly of the clean reads yielded 76,717 expressed transcripts (unigenes), including 13,874 novel genes, with over 78.83% of clean reads mapping to the reference genome. GO analysis assigned 38,655 unigenes (50.39%) to functional categories, with the most abundant subcategories being “metabolic process” (Biological Process), “cellular anatomical entity” (Cellular Component), and “binding” (Molecular Function) (**Supplementary Figure S1**). KEGG pathway annotation assigned 36,468 unigenes (47.54%) to five categories, including 22 subcategories; those with the highest gene representation included “global and overview maps”, “carbohydrate metabolism”, and “translation” (**Supplementary Figure S2**). PCA confirmed clear separation between the transcriptomes of AS and AA (**Supplementary Figure S3**), which confirms the high quality of sampling, sequencing, and gene expression quantification in this study.

**Figure 2:**
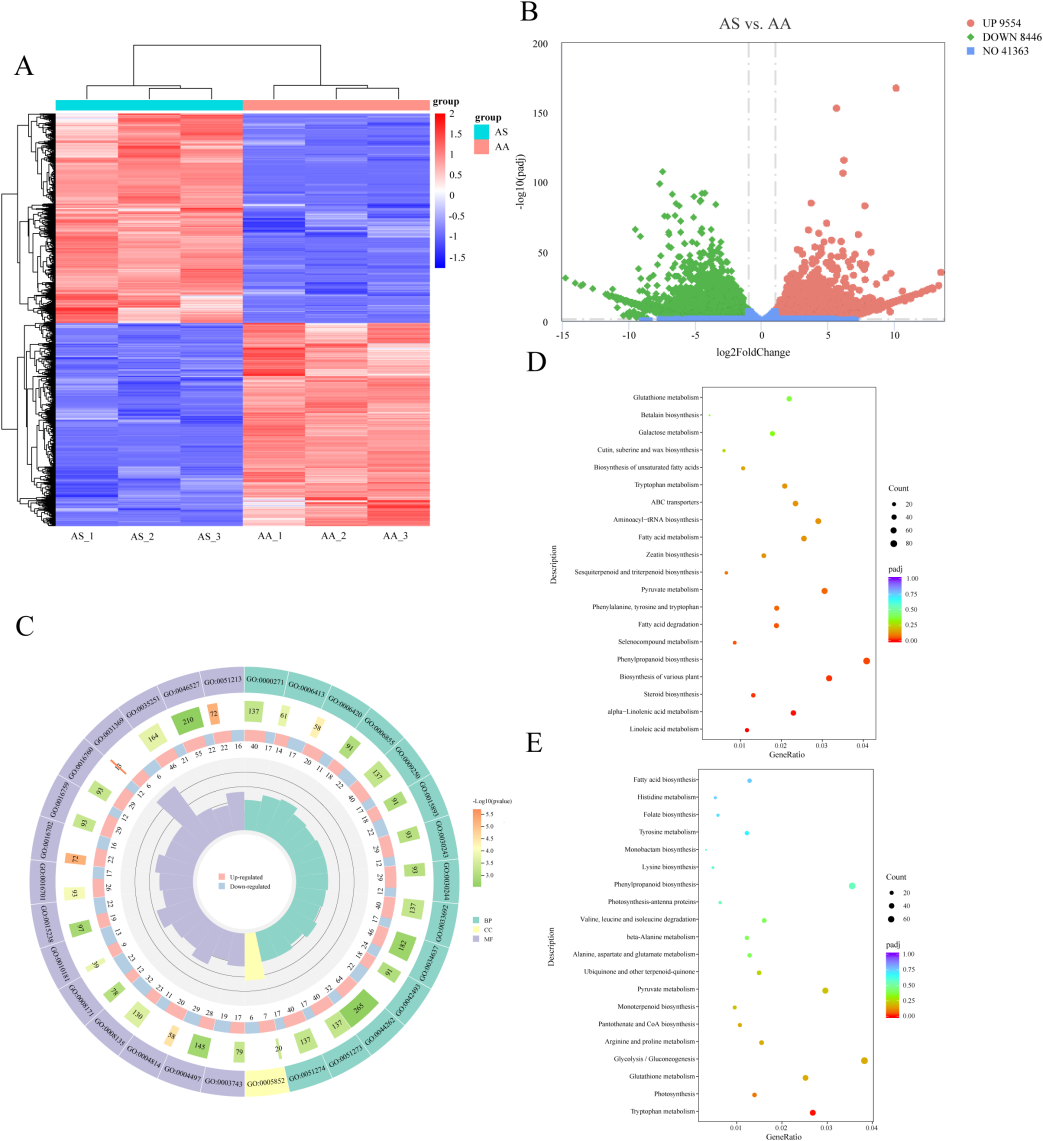
Functional classification of DEGs. **(A)** Hierarchical clustering heatmap of DEGs. **(B)** Volcano plot of DEGs. **(C)** Comparison of the distribution of DEGs at the GO level. KEGG enrichment analysis of upregulated **(D)** and downregulated **(E)** DEGs in AS.

The comparative analysis between the AS and AA groups identified 9,554 upregulated and 8,446 downregulated DEGs in AS relative to AA (**Figures 2A, B**). This substantial set of DEGs likely underlies the observed differences in secondary metabolism between the two species. Among these DEGs, 4,656 were annotated with GO terms, with the most enriched subcategories in BP, CC, and MF being “arginyl-tRNA aminoacylation (GO:0006420)”, “eukaryotic translation initiation factor 3 complex (GO:0005852)”, and “translation initiation factor binding (GO:0031369)”, respectively (**Figure 2C**). To specifically investigate the molecular basis of secondary metabolic differences, we conducted KEGG enrichment analysis on the DEGs. This analysis revealed significant enrichment (*p* < 0.05) of DEGs in pathways directly related to secondary metabolism. Notably, the “phenylpropanoid biosynthesis” pathway was significantly enriched in both upregulated (80) and downregulated (66) gene sets (**Figures 2D, E**). Among the 18 significant pathways enriched for upregulated DEGs in AS, “biosynthesis of various plant secondary metabolites” (62), “linoleic acid metabolism” (23), “flavone/flavonol biosynthesis” (9), and “sesquiterpenoid/triterpenoid biosynthesis” (13) were prominent (**Figure 2D**). For downregulated DEGs in AS (12 significant pathways), the most enriched pathway was “glycolysis/gluconeogenesis” (71), while “monoterpenoid biosynthesis” (18) and “terpenoid backbone biosynthesis” (29) were also enriched (**Figure 2E**). Analysis of the top 20 most significantly enriched KEGG pathways further underscored distinct regulatory mechanisms within phenylpropanoid metabolism (“phenylpropanoid biosynthesis”) and terpenoid metabolism (“monoterpenoid biosynthesis” and “sesquiterpenoid/triterpenoid biosynthesis”) **(Figure S4**). Collectively, these transcriptomic analyses identified extensive DEGs and revealed significant alterations in key secondary metabolic pathways, particularly in phenylpropanoid and terpenoid biosynthesis, providing crucial insights into the molecular mechanisms responsible for the divergent secondary metabolite profiles between *A. stolonifera* and *A. argyi*.

### 3.3 Volatile terpenoids reveals metabolite changes between *A. stolonifera* and *A. argyi*

**Figure 3:**
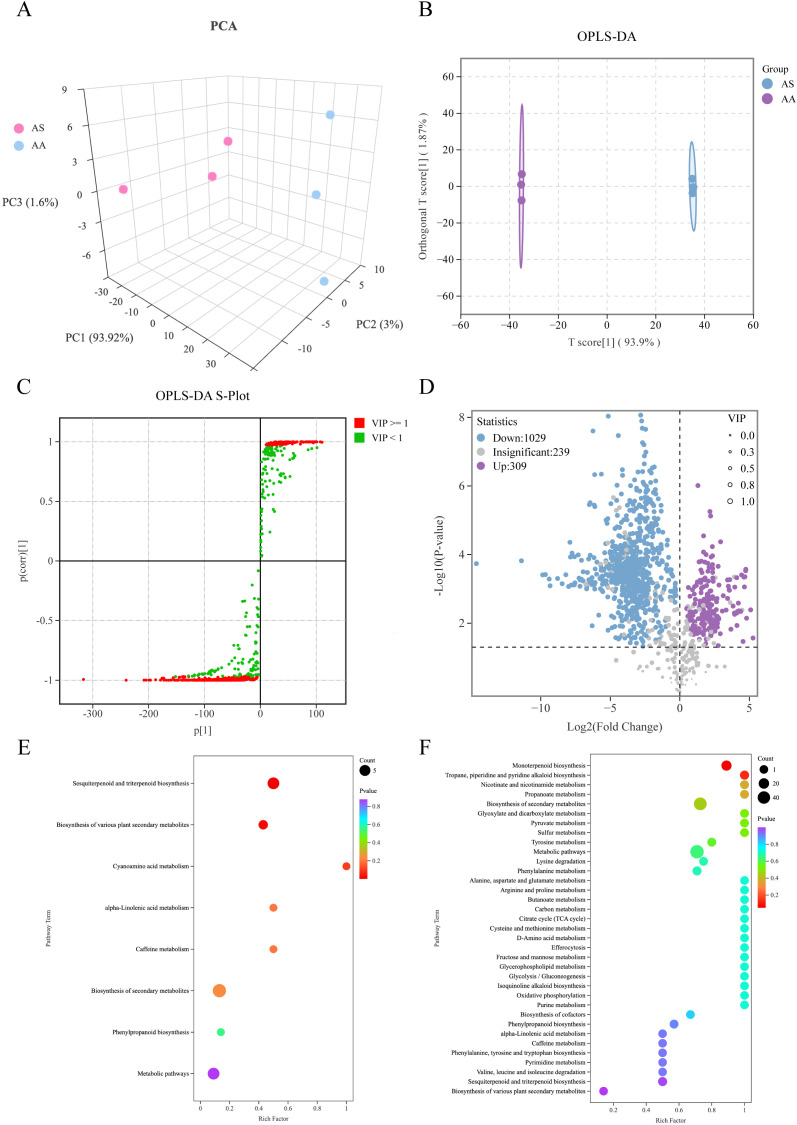
Multivariate statistical analysis of the DAMs based on HS-SPME-GC-MS. **(A)** Three-dimensional score plots of PCA, **(B)** OPLS-DA, **(C)** S-plot of OPLS-DA, **(D)** Volcano plot of DAMs, **(E)** Enrichment analysis of KEGG using upregulated DAMs, **(F)** Enrichment analysis of KEGG using downregulated DAMs in AS.

To comprehensively profile the metabolomic differences between AS and AA, we conducted a metabolic profiling analysis of VOCs using HS-SPME-GC-MS. An initial assessment of the total ion chromatograms (TICs) revealed strong analytical signals, high peak capacity, and good retention time reproducibility across all samples, indicating robust instrument performance (**Supplementary Figure S5**). A total of 1,577 metabolites were detected across all samples and categorized into 15 distinct groups. Terpenoids represented the most abundant class, accounting for 22.32%, followed by esters at 18.14%. Multivariate analysis utilizing PCA and OPLS-DA effectively segregated the samples into two distinct clusters corresponding to AS and AA, underscoring the significant metabolic divergence between *A. stolonifera* and *A. argyi* (**Figures 3A-C**).

Employing both single- and multidimensional analytical approaches, we identified a total of 1,338 DAMs, of which 1,029 were significantly downregulated and 309 were significantly upregulated in AS compared to AA (**Figure 3D**). The top 30 DAMs exhibiting the highest absolute value of Log_2_FC were predominantly terpenoids (**Supplementary Table S3**). KEGG pathway enrichment analysis of the DAMs (**Figures 3E, F**) revealed significant alterations in specific metabolic pathways. Terpenoids constituted the dominant class among both the upregulated and downregulated DAMs, suggesting their key roles in differentiating the species. The upregulated DAMs in AS were extremely significantly enriched (*p* < 0.01) in “sesquiterpenoid/triterpenoid biosynthesis” (6) and significantly enriched (*p* < 0.05) in “biosynthesis of various plant secondary metabolites” (8) (**Figure 3E**). Conversely, the downregulated DAMs in AS were significantly enriched (*p* < 0.05) in “monoterpenoid biosynthesis” (17) (**Figure 3F**). Notably, this pattern of terpenoid metabolite enrichment aligns remarkably well with the transcriptomic data, which showed significant enrichment of DEGs in the corresponding terpenoid backbone and branch pathways (**Figures 2D, E**). Specifically, the upregulation of sesquiterpenoid/triterpenoid DAMs corresponds to the enrichment of upregulated DEGs in pathways such as “sesquiterpenoid/triterpenoid biosynthesis”, while the downregulation of monoterpenoid DAMs correlates with the observed downregulation of DEGs in “monoterpenoid biosynthesis” pathways. These findings strongly support the hypothesis that the differential accumulation of terpenoids in their biosynthetic pathways is a major contributor to the metabolic distinction between *A. stolonifera* and *A. argyi*.

### 3.4 Non-volatile flavonoids and lignin-related metabolites reveal metabolite changes between *A. stolonifera* and *A. argyi*

**Figure 4:**
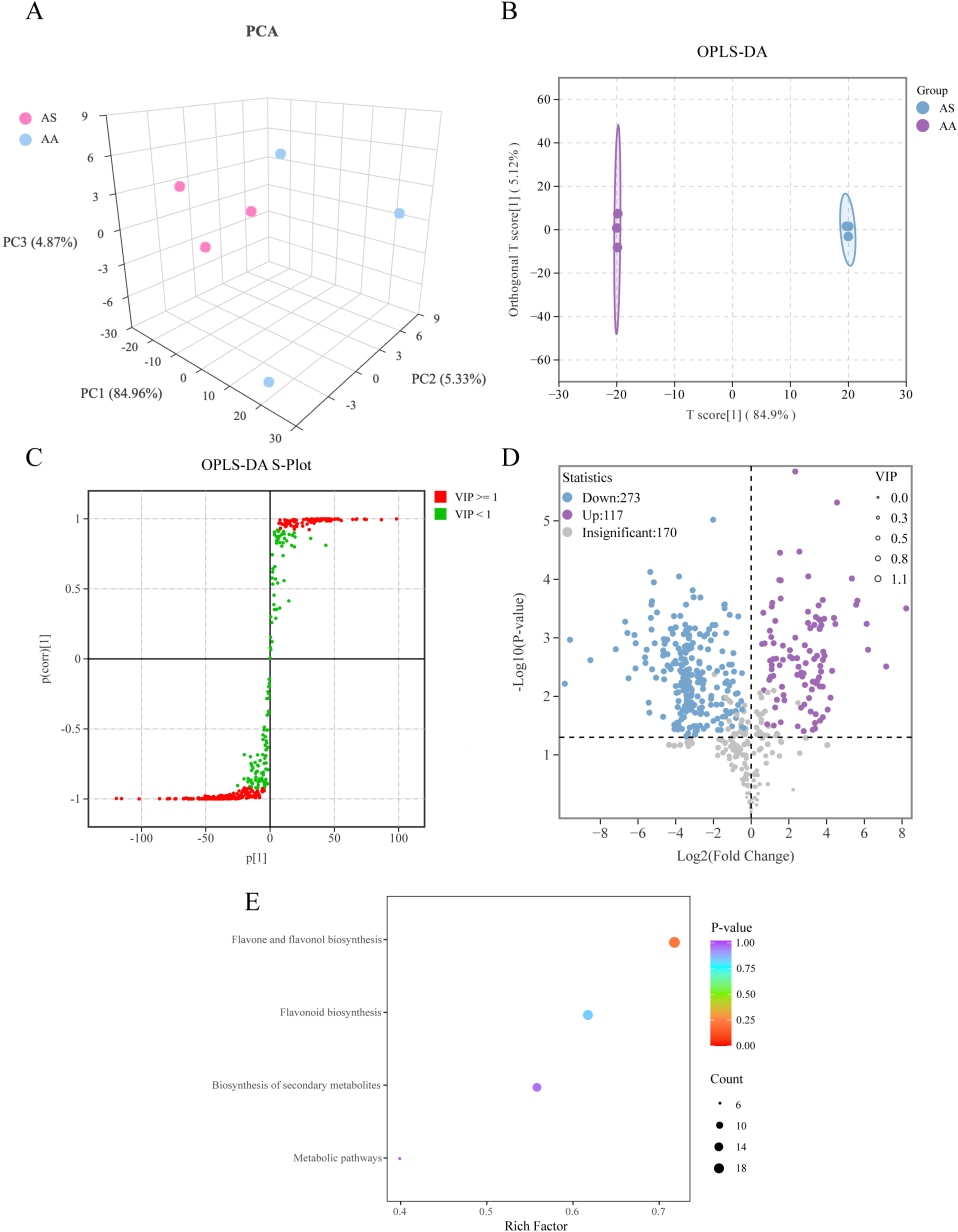
Multivariate statistical analysis of the DSMs based on UPLC-ESI-MS. **(A)** Three-dimensional score plots of PCA, **(B)** OPLS-DA, **(C)** S-plot of OPLS-DA, **(D)** Volcano plot of DAMs. **(E)** Enrichment analysis of KEGG of DAMs.

To investigate secondary metabolic divergence indicated by transcriptomic data, we performed targeted UPLC-ESI-MS analyses focusing on flavonoids and lignin biosynthesis intermediates. The assessment of base peak chromatograms confirmed the robustness of the instrument’s performance (**Supplementary Figure S6**). A total of 560 flavonoid metabolites were identified (417 neg and 143 pos), with flavones (179) and flavonols (130) constituting the predominant subclasses. PCA and OPLS-DA clearly separated AS and AA (**Figures 4A–C**), confirming substantial inter-species metabolic differentiation, as observed in the VOCs metabolomics and transcriptomics. We identified 390 DAMs in the flavonoid dataset, of which 273 were downregulated and 117 upregulated in AS (**Figure 4D**). Among the top 30 DAMs ranked by absolute value of Log2FC, flavones and flavonols showed the most pronounced alterations, with the majority being downregulated in AS (**Supplementary Table S4**). KEGG enrichment highlighted “flavone/flavonol biosynthesis” (21) and “flavonoid biosynthesis” (18) as the most significantly enriched pathways (**Figure 4E**), corroborating transcriptomic findings (**Figure 2**). This analysis illustrates that the predominantly downregulated accumulation of flavones and flavonols in AS significantly contributes to the metabolic differentiation between *A. stolonifera* and *A. argyi*.

**Figure 5:**
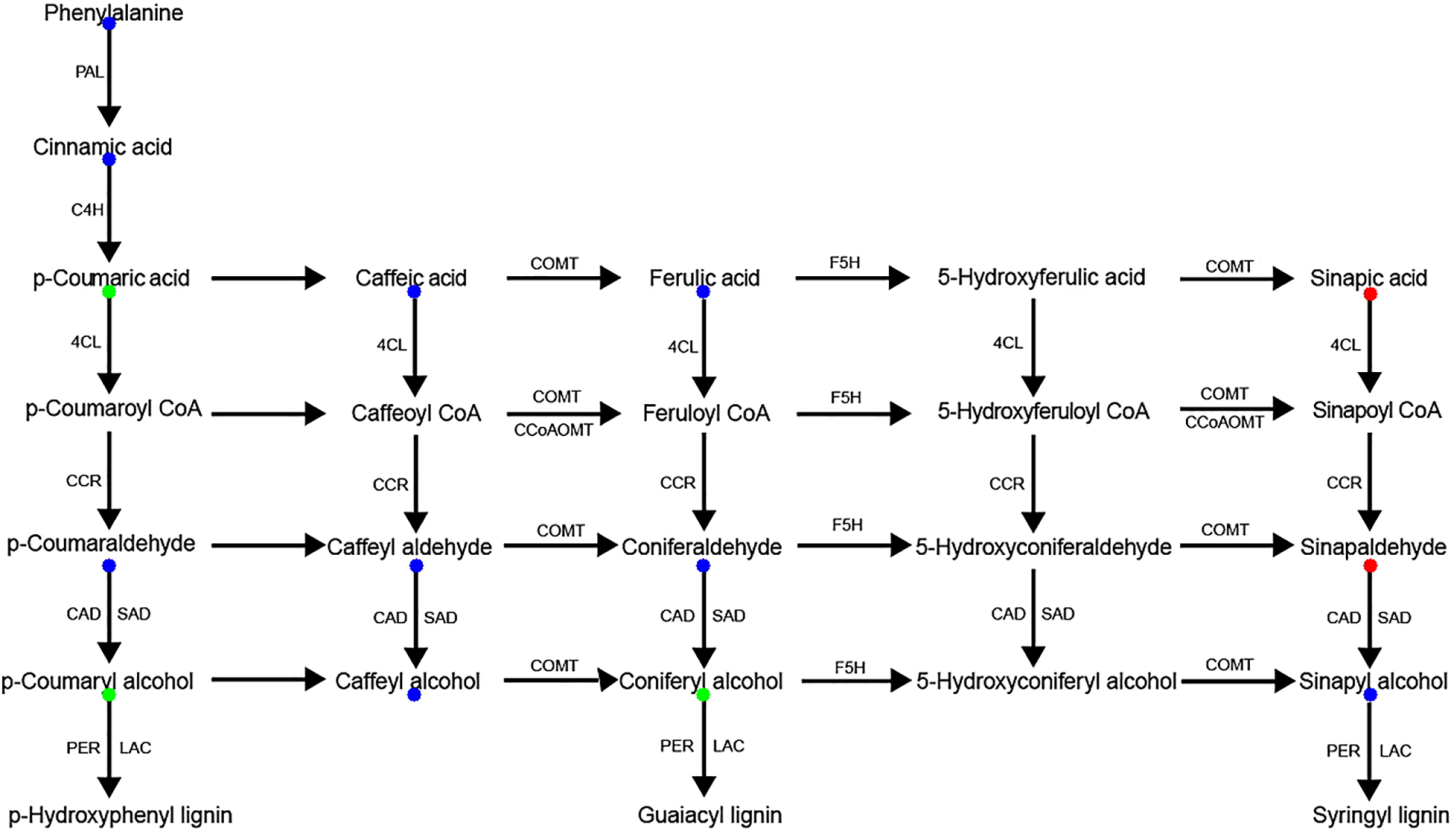
Lignin biosynthetic pathway in AS and AA. Intermediate metabolites detected with no significant difference between AS and AA are marked in blue. Red/green represents up/down-accumulated metabolites in the AS.

Complementary analysis of the lignin biosynthesis pathway identified 14 key intermediate metabolites, among which 5 DAMs were detected. Sinapic acid and sinapinaldehyde, precursors of S-lignin, were significantly upregulated in AS compared to AA, whereas p-coumaric acid, coniferyl alcohol, and p-coumaryl alcohol, precursors of G- and H-lignin, were significantly downregulated (**Figure 5**). These results suggest species-specific lignin subunit composition and illustrate coordinated regulation of phenylpropanoid-derived metabolism. Collectively, our UPLC-ESI-MS data delineate distinct flavonoid and lignin biosynthesis profiles between the two species, providing metabolite-level evidence.

### 3.5 Correlation analysis between the transcriptome and metabolome data

**Figure 6:**
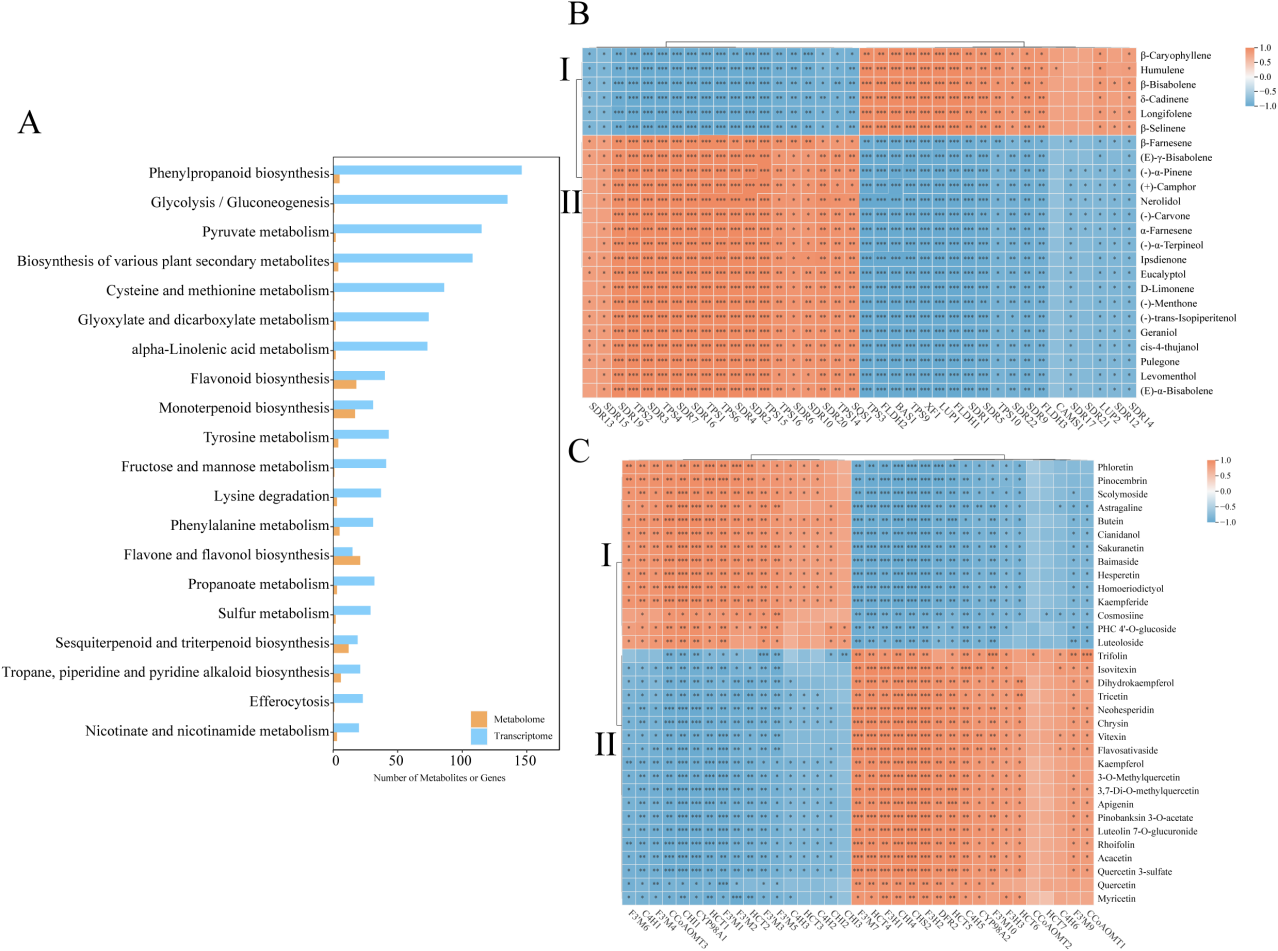
Co-enrichment analysis and correlation analysis of DAMs and DEGs shared in AS and AA. **(A)** Co-enrichment analysis of DAMs and DEGs, **(B)** Correlation analysis of the terpenoid-related DEGs and DSMs, **(C)** Correlation analysis of the flavonoid-related DEGs and DSMs. *pvalue<0.05;**pvalue<0.01; ***pvalue<0.001.

To elucidate the regulation of secondary metabolism, integrated KEGG enrichment analysis revealed 33 pathways co-enriched in DEGs and DAMs. **Figure 6A** highlighted the top 20 pathways with the largest number of DAMs. Among these pathways, flavone/flavonol biosynthesis, flavonoid biosynthesis, monoterpenoid biosynthesis, and sesquiterpenoid/triterpenoid biosynthesis exhibited the highest numbers of DAMs. Notably, sesquiterpenoid/triterpenoid biosynthesis, monoterpenoid biosynthesis, terpenoid backbone biosynthesis, and flavone/flavonol biosynthesis were significantly enriched in the transcriptomic data, indicating their central roles in the differences observed between *A. stolonifera* and *A. argyi*. Specifically, 50 DEGs and 24 DAMs were identified as being associated with the terpenoid-related biosynthesis pathway, while 55 DEGs and 39 DAMs were linked to the flavonoid-related biosynthesis pathway.

Crucially, Pearson correlations were computed using DEGs and DAMs, as visualized in **Figures 6B, C**. This analysis focused on terpenoid-related and flavonoid-related biosynthesis pathways, with a significance threshold set at *p* < 0.01 and |r| > 0.9. Among the findings, 24 terpenoids demonstrated significant correlations with 30 DEGs, while 33 flavonoids were significantly correlated with 27 DEGs. The co-expression analysis revealed that terpenoid metabolites could be distinctly categorized into two groups: Category I, which includes 6 sesquiterpenoids, and Category II, which comprises of 5 sesquiterpenoids and 13 monoterpenoids. Notably, 21 DEGs were positively correlated with Category I DAMs and negatively correlated with Category II DAMs (**Figure 6B**). Similarly, flavonoid-related metabolites were classified into two categories: Category I, consisting of 4 flavanones, 3 flavones, 3 flavonols, 3 chalcones and 1 flavanol, and Category II, which includes 9 flavones, 7 flavonols, 2 flavanonols and 1 flavanone. A total of 16 DEGs were positively correlated with Category I DAMs (**Figure 6C**). Within each category, metabolites exhibited positive correlations, while those from different categories typically displayed negative correlations. Collectively, we hypothesize that species-specific divergence is mechanistically linked to the differential regulation of terpenoid-related and flavonoid-related biosynthesis.

### 3.6 Identification of unigenes related to terpenoid-related, and flavonoid-related biosynthesis pathways

Our results, obtained through the integration of transcriptomics and metabolomics, indicate significant differences in the expression of genes related to terpenoid and flavonoid biosynthesis between the two species. The mevalonate (MVA) and methylerythritol phosphate (MEP) pathways are central to terpenoid biosynthesis, generating isoprenoid precursors including isopentenyl diphosphate (IPP) and dimethylallyl diphosphate (DMAPP). To investigate the potential functions of genes involved in terpenoid backbone biosynthesis in leaves, which serve as the primary storage organ for essential oils, we identified candidate genes encoding enzymes within the MVA and MEP pathways from RNA-seq data. Transcriptomic analysis revealed 48 DEGs associated with terpenoid backbone biosynthesis, of which 19 unigenes exhibited upregulation in AS compared to AA. Within the MVA pathway, acetyl-CoA C-acetyltransferase (AACT), hydroxy methylglutaryl CoA reductase (HMGR), and phosphomevalonate kinase (PMK) showed species-specific regulation. Notably, the expression of *AACT1–4* was markedly elevated in AS, corresponding to a 2.3- to 5.0-fold increase relative to AA. In contrast, genes associated with the MEP pathway, such as 1-deoxy-D-xylulose-5-phosphate synthase (DXS) and reductoisomerase (DXR), displayed preferential expression in AA.

**Figure 7:**
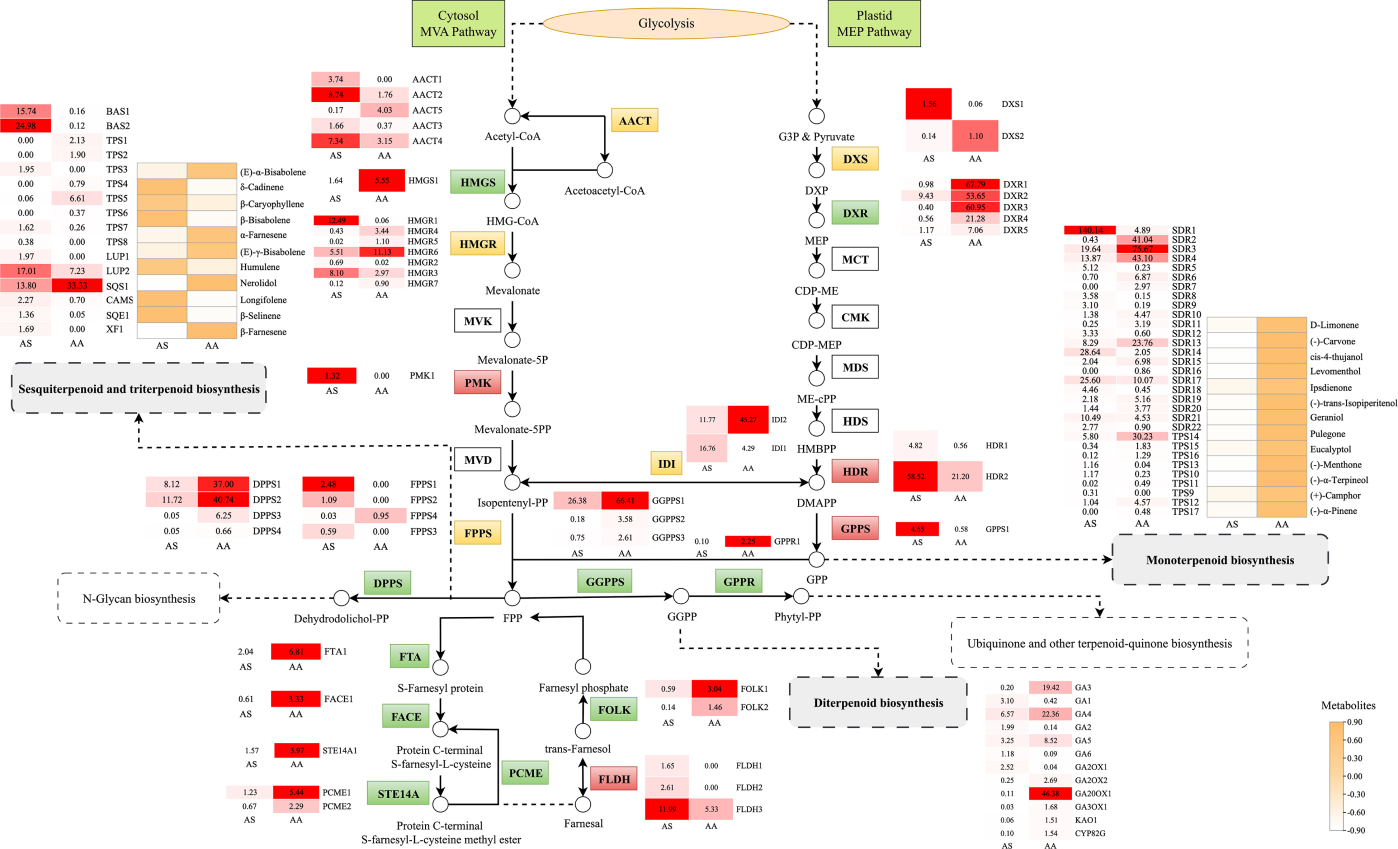
Metabolic pathway map of the terpenoid-related biosynthesis pathways. Small squares and circles indicate the expressed levels of unigenes/metabolites, and red/green/yellow graphics indicates up/down/both up- and downregulated genes or metabolites in the AS group.

Downstream terpenoid synthesis revealed nuanced regulatory patterns. Farnesyl diphosphate synthase (FPPS) isoforms (*FPPS1-3*) exhibited statistically significant, albeit low-abundance, upregulation in AS, indicating a subtle enhancement of sesquiterpenoid precursor production. Additionally, four additional genes (*FLDH1–3* and *GPPS1*) demonstrated AS-upregulated expression linked to specialized terpenoid diversification. Sesquiterpenoid/triterpenoid biosynthesis pathways contained 19 DEGs, predominantly upregulated in AS. In contrast, monoterpenoid and diterpenoid biosynthesis involved 31 and 12 DEGs respectively, with 18 and 8 unigenes upregulated in AA. Metabolomic validation confirmed species-specific accumulation. Notably, all 13 DAMs in monoterpenoid biosynthesis significantly increased in AA, with (-)-carvone, (-)-menthone, and *cis*-4-thujanol exhibiting 79.07-, 28.62-, and 24.57-fold higher contents respectively (**Figure 7**). Furthermore, 11 DAMs associated with sesquiterpenoid and triterpenoid biosynthesis were identified, predominantly upregulated in AS.

**Figure 8:**
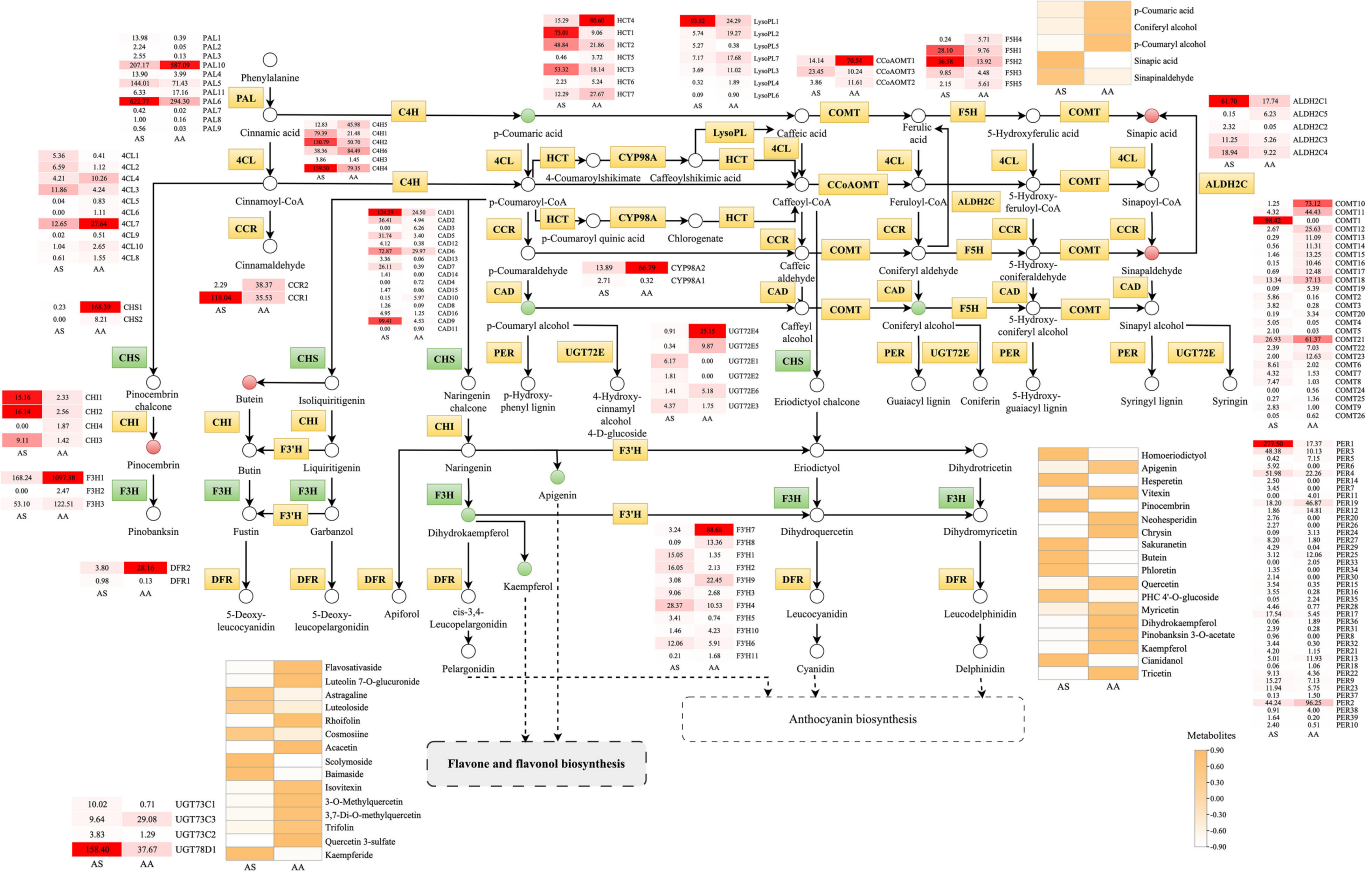
Metabolic pathway map of the flavonoid-related biosynthesis pathways. Small squares and circles indicate the expressed levels of unigenes/metabolites, and red/green/yellow graphics indicates up/down/both up- and downregulated genes or metabolites in the AS group.

Through combined transcriptomic and metabolomic profiling, along with KEGG pathway mapping, we reconstructed the species-divergent regulation of flavonoid biosynthesis in AS and AA. The phenylpropanoid pathway served as the central biosynthetic node, supplying precursors for both flavonoid and lignin production. In this study, we identified 145, 40, and 15 DEGs involved in phenylpropanoid, flavonoid, and flavone/flavonol biosynthesis, respectively, of which 80, 19, and 9 DEGs were significantly upregulated (*p* < 0.05) in AS. Correspondingly, 5, 18, and 15 metabolites were associated with phenylpropanoid, flavonoid, and flavone/flavonol biosynthesis (**Figure 8**). We identified distinct expression patterns for 39 peroxidases (PERs), 26 acetylserotonin O-methyltransferases (COMTs), 16 cinnamyl-alcohol dehydrogenases (CADs), 10 4-coumarate-CoA ligases (4CLs), 7 spermidine hydroxycinnamoyl transferases (HCTs), 7 lysophospholipases (LysoPLs), 6 cinnamate-4-hydroxylases (C4Hs), 6 UDP-glucosyl transferases 72E (UGT72Es), 5 aldehyde dehydrogenases (ALDH2Cs), 5 ferulate-5-hydroxylases (F5Hs), 3 caffeoyl-CoA O-methyltransferases (CCoAOMTs), 2 cytochrome P450 family 98 subfamily A8s (CYP98As), and 2 cinnamoyl-CoA reductases (CCRs) between the two species. This metabolic profile aligns with the observed transcriptomic differences, particularly the downregulation of key precursors for G-lignin (coniferyl alcohol) in AS, in conjunction with the upregulation of intermediates associated with S-lignin synthesis (sinapic acid, sinapinaldehyde). These monolignols are subsequently polymerized to form the complex lignin polymer.

The early steps of the flavonoid pathway exhibited complementary regulation among species. Chalcone synthase (CHS) genes showed significantly higher expression in AA, facilitating the accumulation of naringenin chalcone. In contrast, chalcone isomerase (CHI) isoforms displayed species-specific induction, with CHI2 expression increasing 6.3-fold, thereby enhancing the conversion to naringenin. Downstream hydroxylation modifications indicated pathway specialization. Flavanone 3-hydroxylase (F3H) expression increased by an average of 4.4-fold in AA, catalyzing the conversion of flavanones into flavanonols. The expression of flavonoids 3’-monooxygenase (F3’H) isoforms demonstrated divergent regulation: *F3’H7–11* expression rose between 2.9 and 148.4-fold in AA, while *F3’H1–6* expression by 2.0 to 11.1-fold in AS, facilitating the conversion of isoflavones into flavonols. Glycosylation processes played a crucial role in determining the final metabolite speciation. A total of 3 UDP-glycosyltransferases (UGT) 73Cs and 1 UGT78D were identified, with their expression levels varying between the leaves of the two *Artemisia* species. Metabolomic validation corroborated this divergence. Among 15 DAMs in flavone/flavonol biosynthesis, 9 compounds preferentially accumulated in AS, including kaempferide, while 6 metabolites enriched in AA, exemplified by naringenin. Therefore, the differential expression of UGT73Cs and UGT78D may be a critical factor directly influencing the type of flavone/flavonol synthesized.

### 3.7 qRT-PCR validation of DEG expression

**Figure 9:**
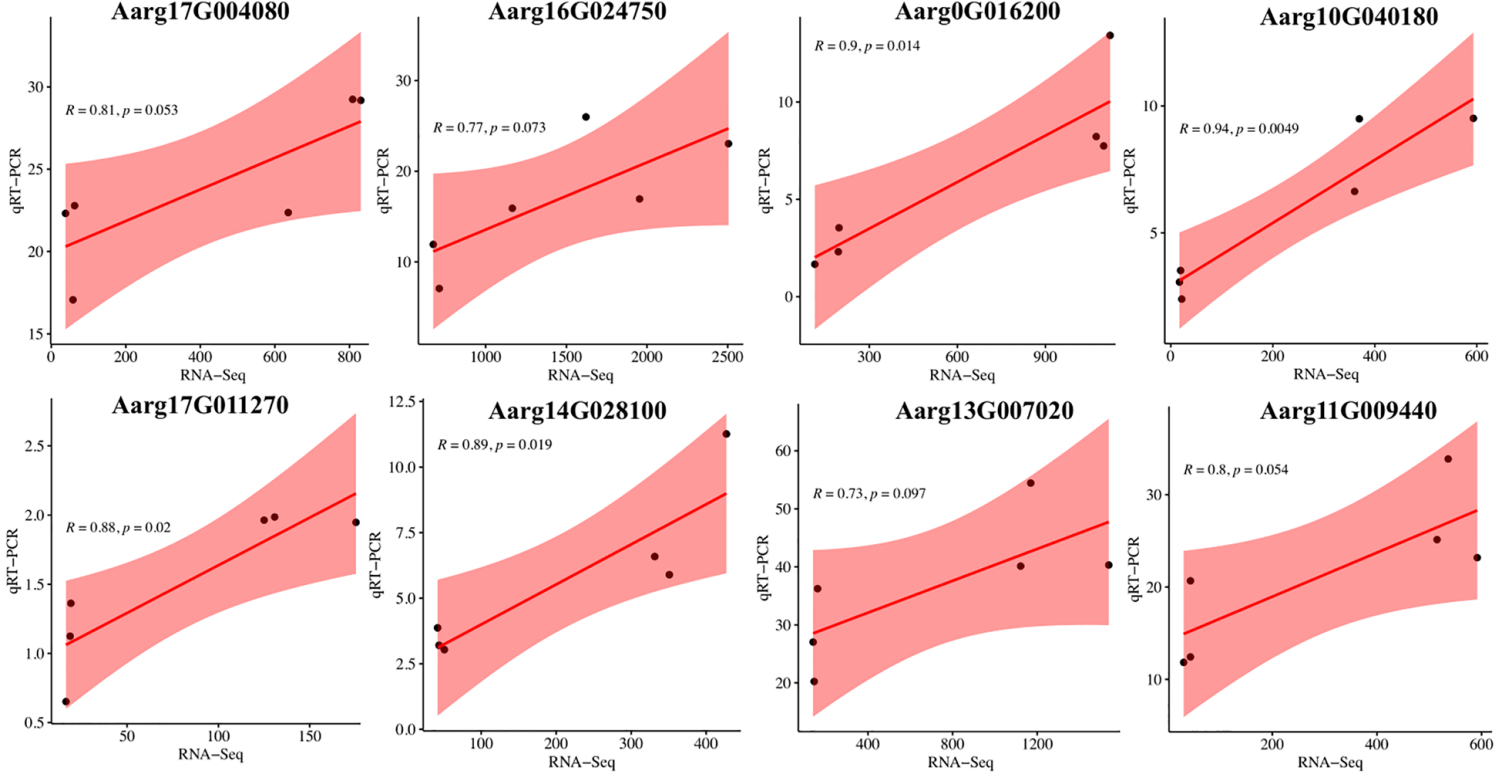
Scatter plot of Pearson correlation coefficient between RNA-Seq and qRT-PCR.

To validate the reliability and robustness of our transcriptome dataset, we conducted an independent verification by quantifying the expression patterns of eight DEGs using qRT-PCR. The expression profiles of these DEGs were consistent with those obtained from RNA sequencing analysis (**Figure 9**). This robust correlation establishes a reliable resource for elucidating species-specific divergence in specialized metabolite pathways between *A. stolonifera* and *A. argyi*, particularly in the terpenoid and flavonoid metabolic networks.

## 4 Discussion

Currently, *A. argyi* is officially documented in the Chinese Pharmacopoeia and is extensively cultivated across China for medicinal, food, and moxibustion applications, owing to its well-characterized efficacy and intensely aromatic properties. *A. stolonifera* exhibits a mild and delicate aroma, despite documented historical use, remains markedly underexplored and underutilized in modern applications, primarily due to insufficient comparative studies of its phytochemical aspects. Our integrated metabolomic platform characterized 2,153 metabolites (1,579 via GC-MS and 574 via UPLC- ESI (-QTRAP) -MS) with clear definitions. Among these, 1,657 metabolites were identified as DAMs, showing pronounced divergence between *A. stolonifera* and *A. argyi* (**Supplementary Figures S2**, **S5**). This represents an expansion in metabolite coverage compared to prior comparative studies. Li et al. (2023) employed HS-SPME-GC-MS to profile essential oils in *A. stolonifera* (59 volatile compounds) and *A. argyi* (64 volatile compounds). Luo et al. (2020a) utilized UPLC-Q-TOF-MS to analyze the chemical components of leaf and floss extracts from *A. stolonifera* and *A. argyi* sourced from different locations, identifying 18 compounds. Crucially, our non-targeted UPLC-ESI-MS and targeted UPLC-ESI-QTRAP-MS approaches, complementing the HS-SPME-GC-MS dataset, enabled unprecedented resolution of polar bioactive compounds, establishing a foundational framework for elucidating species-specific metabolic networks. Our results delineated distinct chemotypes: *A. stolonifera* accumulates sesquiterpenoids, while *A. argyi* enriches in monoterpenoids and flavonoids. The compositional differences identified through chemical analysis elucidate the contrasting olfactory profiles of the two species. *A. stolonifera* exhibits a much milder and more delicate aroma, a sensory characteristic closely linked to its significantly lower abundance of volatile constituents and reduced levels of strong-smelling monoterpenoids such as eucalyptol and camphor. Conversely, *A. stolonifera* contains higher proportions of lighter, less aggressive sesquiterpenoids, which contribute to its subtle and pleasant aroma. These characteristics suggest that *A. stolonifera* may be a suitable material for applications where milder odors are preferred, such as in sensitive-skin moxibustion or light-aroma therapeutics. In stark contrast, *A. argyi* produces a distinctively strong and penetrating aroma, which primarily arises from its high concentrations of monoterpenes. Species-specific metabolic have also been reported in other medicinal genera. In *Coptis* species, interspecific variation in alkaloid profiles directly influences pharmacological potency (Qi et al., 2022), supporting our hypothesis that the metabolic differences observed between *Artemisia* species have significant functional implications.

Field cultivation practices confirm that both *A. argyi* and *A. stolonifera* undergo stem lignification during development, a process supported by the positive correlation between lignin content and stem strength (Nan et al., 2022). Metabolomic profiling revealed higher accumulation of lignin pathway intermediates (p-coumaryl alcohols and coniferyl alcohols) in AA, indicating enhanced lignin deposition. Following the synthesis of monolignols (p-coumaryl alcohols, coniferyl alcohols, and sinapyl alcohols) in the cytoplasm, these compounds are transported to the apoplast where they are polymerized with lignin units (S, H and G units) by peroxidase (POD) and laccase (LAC) (Bonawitz and Chapple, 2010; Miao and Liu, 2010; Shah et al., 2017). Transcriptomic analysis further identified 145 DEGs involved in lignin biosynthesis, most exhibiting higher expression in AA, consistent with the metabolite data. In contrast, the lower accumulation of intermediate lignin metabolites in AS suggests that more phenylpropanoid precursors might be channeled to the biosynthesis of volatile terpenoids or aromatic esters, providing a metabolic explanation for its mild and fresh aroma profile. This divergence in phenylpropanoid partitioning provides a biochemical basis for the distinct aroma profiles of the two species.

Terpenoids are a class of important chemicals produced by plants, classified by carbon skeleton size into monoterpenes (C₁₀), sesquiterpenes (C_15_), diterpenes (C₂₀), triterpenes (C₃₀), tetraterpenes (C₄₀), and polyterpenes. Among these, sesquiterpenoids/triterpenoids are primarily biosynthesized via the cytosolic MVA pathway (Dubey et al., 2003). Focusing on terpenoid biosynthesis, structural genes within the MVA pathway exhibited significantly elevated expression (e.g., *AACT1-4*, *HMGR1-3*) in *A. stolonifera*. HMGR, the rate-limiting enzyme that converts HMG-CoA to mevalonate (Rodríguez-Concepción and Boronat, 2002; Rohmer, 2007), positively regulates terpenoid synthesis (Gondet et al., 1992; Zhang et al., 2019), which partially explains the accumulation of sesquiterpenoids observed in *A. stolonifera*. Conversely, genes of the MEP pathway displayed significantly higher expression (e.g., *DXS2*, *DXR1-5*) in *A. argyi* (**Figure 7**). *DXS* and *DXR* are key rate-limiting enzymes (Lange et al., 1998; Takahashi et al., 1998) that enhance the precursor supply for monoterpene biosynthesis (Lichtenthaler et al., 1997). The divergent terpenoid biosynthesis in *A. stolonifera* and *A. argyi* likely stems from evolutionary specialization. Evidence suggests that organellar genomes, in addition to nuclear genes, may influence metabolic differentiation. Mitochondrial genomic studies in *Coptis* species have demonstrated substantial organelle-level variation associated with interspecific metabolic divergence (Zhong et al., 2023), suggesting that cyto-nuclear interactions could similarly contribute to metabolic disparity in *Artemisia*. Transcriptome-metabolome correlation analysis identified key genes (*TPS*, *SQS*, *BAS*, *SDR*, *LUP*, *CAMS*, *SQE*, and *XF*) in terpenoid-related biosynthesis pathways regulating the differential synthesis of terpenoids in *A. stolonifera* and *A. argyi* (**Figure 6B**). Specifically, the enhanced expression of downstream terpenoid backbone biosynthesis genes (e.g., *BAS1*, *LUP2*, *CAMS1*, and *XF1*) in *A. stolonifera* showed a strong positive correlation with its significantly higher accumulation of specific sesquiterpenoids, including δ-cadinene, β-bisabolene, β-caryophyllene, humulene, longifolene, and β-selinene (**Figures 6B**, **7**), indicating an enhanced flux toward sesquiterpenoid precursors. The elevated expression of key downstream genes (e.g., *SDR2-4*, *TPS14*) is closely associated with the preferential accumulation of monoterpenoids, such as eucalyptol and (+)-camphor. The complexity of this metabolic network, with characterized by numerous branches, underscores the necessity for further investigation into the cooperative expression and regulatory mechanisms of each gene. The terpenoids metabolic divergence between *A. stolonifera* and *A. argyi* may extend beyond transcriptional regulation to include post-translational modifications. PRMT5-mediated methylation significantly reshapes protein function and cell metastasis (Fan et al., 2025), suggesting that analogous mechanisms may similarly influence metabolic specialization in *Artemisia* species.

**Figure 10:**
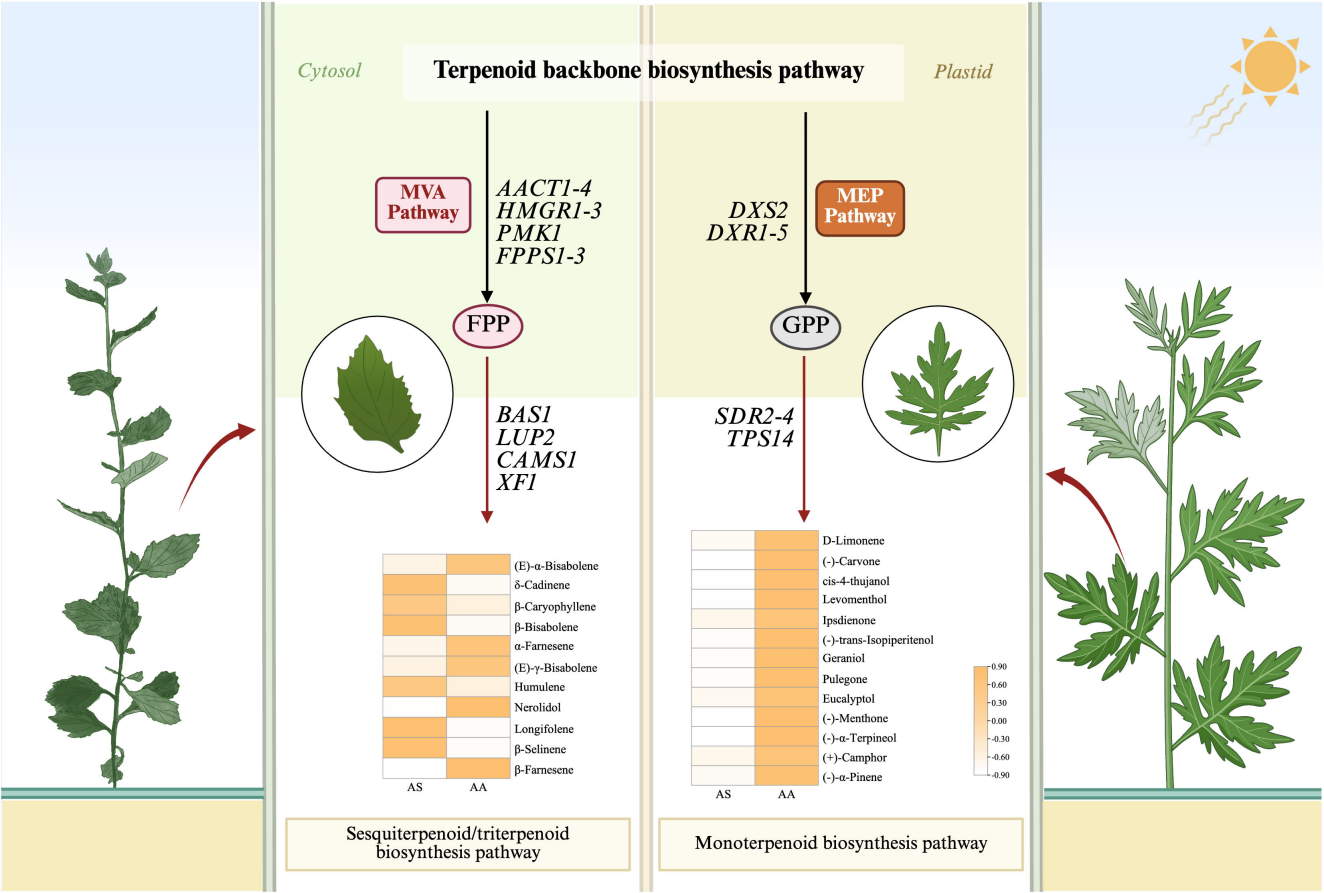
Regulatory network and mechanism of terpenoid biosynthesis in AS and AA.

The differences identified between *A. stolonifera* and *A. argyi* emphasize the need for a deeper understanding of the properties of individual species used in various applications. This perspective echoes recent advances in medicinal plant resource evaluation, including innovative approaches to utilizing non-traditional plant parts as demonstrated in *Coptis* research (Ding et al., 2025). Previous studies have validated that monoterpenes exhibit potent antimicrobial and antioxidant activities, positioning them as ideal natural food preservatives that ensure the microbiological and oxidative stability of food products (Rehman et al., 2021; Kumar et al., 2022). Our metabolomic quantification confirmed that monoterpenes are primarily enriched in *A. argyi* (**Figure 10**), establishing this species as a premium source for natural food preservatives. Additionally, the higher levels of eucalyptol and camphor further highlight its suitability as a promising additive (Yazgan, 2020; Šojić et al., 2021). *A. stolonifera* is characterized by a high abundance of sesquiterpenoids, especially β-caryophyllene. Mechanistic studies demonstrate β-caryophyllene possesses a greater ability to retain in the stratum corneum compared to eucalyptol (**Figure 10**), thereby facilitating its disruptive effect on stratum corneum lipids (Tang et al., 2023). According to Zhang et al. (2024), seven flavonoids (5-desmethylnobiletin, rhoifolin, baicalin, biorobin, nepetin, eriodictyol, and isorhoifolin) and nine terpenoids (bornyl acetate, endo-borneol, L-fenchone, 2-pinen-7-one, caryophyllene, camphor, levomenthol, (+)-β-cedrene, and γ-terpinene) were identified as important biomarkers for tracking the aging time of *A. argyi*. The transformation of these compounds during post-harvest aging is critical for enhancing both the efficacy and safety of *A. argyi*, corroborating the traditional practice of prolonged storage. In contrast, the inherently mild properties of *A. stolonifera* suggest that it may not require extended aging to achieve optimal applicability. This fundamental difference not only indicates distinct post-harvest processing needs for the two species but also positions *A. stolonifera* as a promising resource with advantages for rapid utilization.

Flavonoids, which are low molecular weight secondary metabolites with significant pharmacological value, are predominantly biosynthesized via the phenylpropanoid pathway (Nigam et al., 2019). An analysis of flavonoid biosynthesis in *A. argyi* further demonstrated a significantly elevated expression of core structural genes (e.g., *CHS1-2*, *CHI*, *F3H1-3*) (**Figure 8**), correlating with the notably higher accumulation of specific flavonoids, including chrysin, neohesperidin, and isovitexin. These findings align with previous studies on the flavonoid profiles of *Artemisia* species (Liu et al., 2015; Song et al., 2017), confirming species-specific accumulation. Mechanistically, *CHS* catalyzes the stepwise condensation of 4-coumaroyl-CoA and malonyl-CoA to naringenin chalcone, thereby diverting phenylpropanoid flux toward flavonoids (Winkel-Shirley, 2001; Grotewold, 2006). The *CHS* genes constitute a polygenic family characterized by strong conservation (Park et al., 2020). Subsequent enzymatic conversions drive downstream synthesis: *CHI* catalyzes the isomerization of chalcone to naringenin (Dixon and Paiva, 1995), while the reaction catalyzed by *F3H* converts naringenin to dihydrokaempferol, which is subsequently converted to kaempferol by FLS (Winkel-Shirley, 2001). Thus, the coordinated upregulation of *CHS*, *CHI*, and *F3H* was positively associated with flavonoid synthesis in *A. argyi*. Collectively, these transcriptional changes underlie the significantly higher flavonoid accumulation in *A. argyi* relative to *A. stolonifera*. These biochemical advantages translate into substantial agricultural applications. The flavonoids from *Artemisia* have been shown to improve the growth performance and meat quality of broilers (Yang et al., 2021; Shi et al., 2022). Notably, 0.4 mg/mL of *A. argyi* flavonoids effectively prolongs the storage period of fresh-grade breast meat (Yang et al., 2023). The abundant flavonoid metabolites underlying these applications provide a biochemical basis for future innovations in deep processing beyond traditional moxibustion.

## 5 Conclusion

This study established a comprehensive multi-omics integration of metabolomic and transcriptomic profiles contrasting the historically significant *A. stolonifera* with the pharmacopeial standard *A. argyi*, revealing species-specific divergences. Comparative analysis identified 1,728 DAMs and 18,000 DEGs, demonstrating that species-specific divergence drives transcriptional and metabolic network reorganization in *A. stolonifera* and *A. argyi*. Transcriptomic profiling revealed significant regulatory divergence in terpenoid and flavonoid biosynthesis between the two species. In *A. stolonifera*, the coordinated upregulation of MVA pathway genes and sesquiterpene synthases strongly correlated with sesquiterpenoid accumulation. Conversely, *A. argyi* exhibited preferential induction of MEP pathway genes, monoterpenoid biosynthesis genes, and flavonoid biosynthesis genes, which are associated with monoterpenoid and flavonoid enrichment. These pronounced metabolic and transcriptional differences underscore the necessity for evaluating of *A. stolonifera*’s distinct phytochemical profile, particularly concerning its equivalence to *A. argyi* for medicinal applications reliant on monoterpenoids or specific flavonoids. Furthermore, while we elucidated novel molecular regulatory networks governing the biosynthesis of terpenoids and flavonoids, the potential regulatory characteristics still required systematic characterization. Our research team is currently investigating the precise functions of specific genes in *A. stolonifera* to decipher their mechanistic contributions to terpenoid biosynthesis. Our findings established a foundational framework for future research, including the functional validation of candidate genes involved in *A. stolonifera*’s terpenoid and flavonoid biosynthesis, which will inform strategies for its precision breeding and sustainable utilization in TCM.

## Data Availability

The datasets presented in the study are publicly available. This data can be found here: NCBI SRA repository under the project number PRJNA1363732.
