# Supplementary material for: Transcriptome and metabolome analyses revealed the main profiles contributing the mild aroma characteristics of Artemisia stolonifera
Source: Front Plant Sci. 2025 Nov 26;16:1713657. doi: 10.3389/fpls.2025.1713657 (PMC12689896; doi:10.3389/fpls.2025.1713657)
Supplement: Supplementary file 1 [file DataSheet1.docx]

**Transcriptome and Metabolome Analyses Revealed the Main Profiles Contributing the Mild Aroma Characteristics of *Artemisia stolonifera***

**Ye Cao^1,2^, Yan Ren^1,2^, Ye Wang^1,2*^, Hui Li^1,2,3*^**

^1^Jiangxi Key Laboratory for Sustainable Utilization of Chinese Materia Medica Resources, Institute of Traditional Chinese Medicine Health Industry, China Academy of Chinese Medical Sciences, Nanchang 330115, China;

^2^Jiangxi Institute of Traditional Chinese Medicine Health Industry, Nanchang 330115, China;

^3^Institute of Chinese Materia Medica, China Academy of Chinese Medical Sciences, Beijing 100700, China

*** Correspondence:**

Corresponding Author

Ye Wang and Hui Li: Building 3, Public Research and Development Center, Traditional Chinese Medicine Science and Technology Innovation City, Xinqizhou Management Office, Xinjian District, Nanchang City, Jiangxi Province, 330115;

Tel.: 0791-83068969

E-mail: ywang@itcmhi.ac.cn (Ye Wang); HLi202201@126.com (Hui Li)

***Supplementary materials***

**
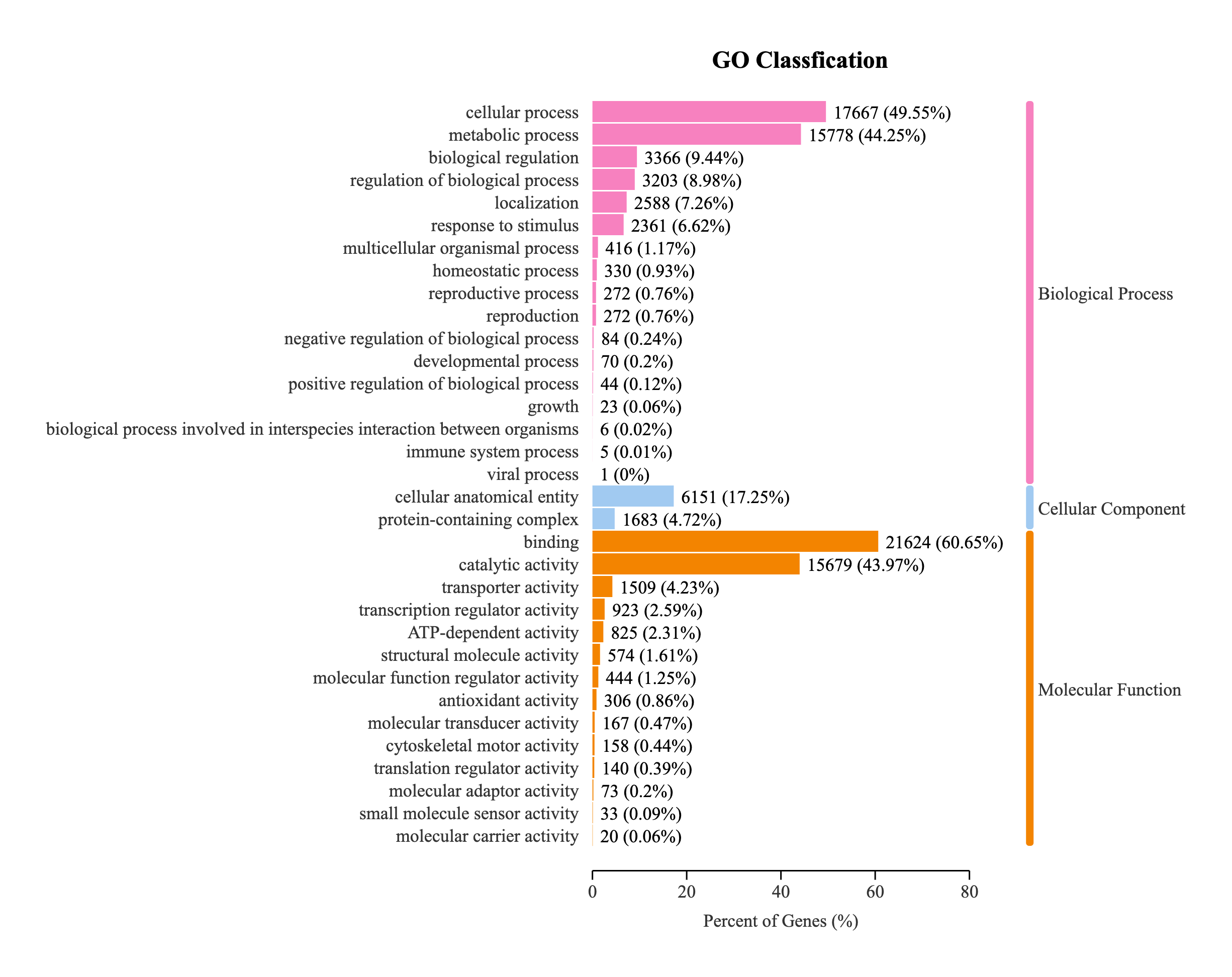
**

**Supplementary Figure 1.** GO analysis of RNA sequencing. The *x*-axis represents the percent of genes, the *y*-axis represents GO functional classification, and the number on the right side of the column represents the number of genes annotated to the pathway.

**Supplementary Figure 2.** KEGG annotation statistics of RNA sequencing. The *x*-axis represents the percent of genes, the *y*-axis represents the name of the pathway, and the number on the right side of the column represents the number of genes annotated to the pathway.

**Supplementary Figure 3.** Principal component analysis of RNA sequencing. The *x*- and *y*-axes represent the first (PC1) and second (PC2) principal components, respectively.

**
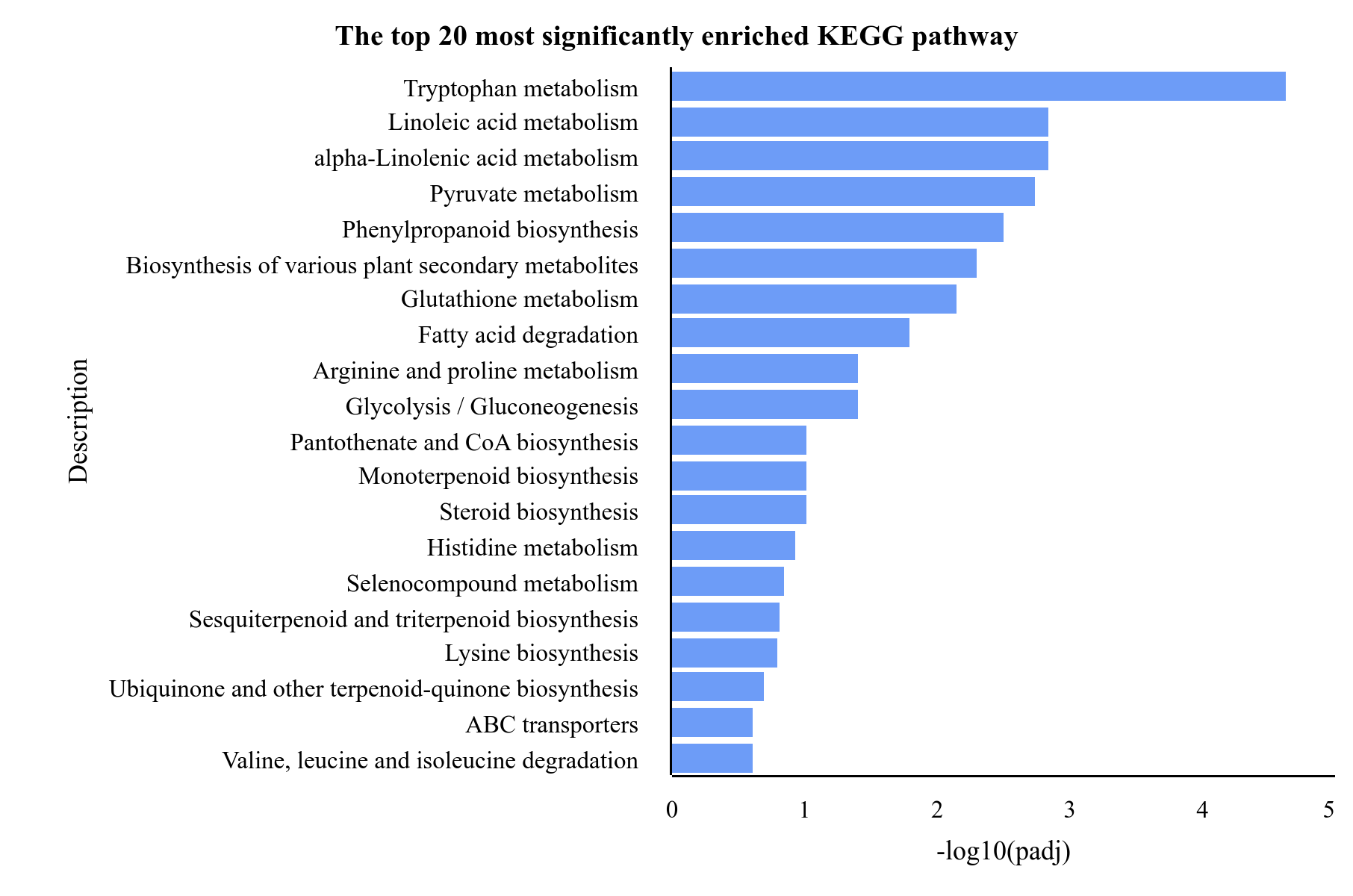
**

**Supplementary Figure 4.** The top 20 most significantly enriched KEGG pathways of RNA sequencing. The *x*- and *y*-axes represent the -log_10_ (padj) and pathway term, respectively.

**
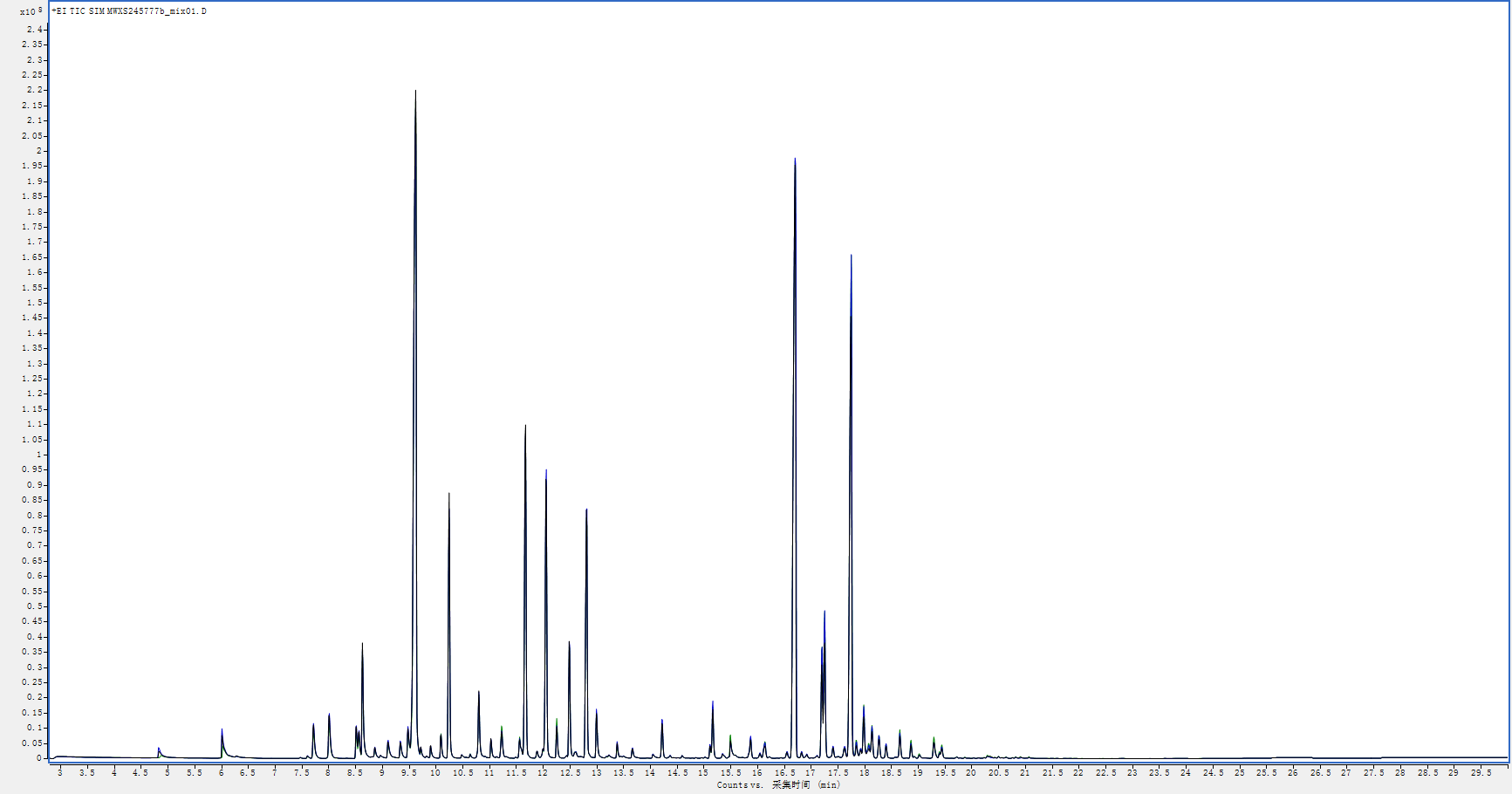
**

**Supplementary Figure 5.** TIC overlay of QC sample mass spectrometry detection based on HS-SPME-GC-MS. The *x*-axis represents retention time, and the *y*-axis represents intensity.

**Supplementary Figure 6.** QC samples total ion chromatograms obtained in negative or positive ion mode based on UPLC-ESI-MS. The *x*-axis represents retention time, and the *y*-axis represents intensity.

**Supplementary Table 1.** The list of qRT-PCR primers.

| **Unigene** | **Forward primer (5’-3’)** | **Reverse primer (5’-3’)** |
| --- | --- | --- |
| *Aaactin* | ACTGAAGCTCCTTTGAACCC | AGAACAATACCAGTAGTACGTCCAC |
| *Aarg0G016200* | CGGTGGGAAGAAAGGCGGATTC | GGCCACCTGGAGTAGTCTCTAGC |
| *Aarg10G040180* | CGTGTTGGCACCTCTTCCTATCG | CGAGCCGGGTTGATTCCAGTTC |
| *Aarg11G009440* | TGCTAAGAAGTTCGCTGGTGCTG | CCCACAACCTTGTCCACCACTTC |
| *Aarg13G007020* | ATCGTCAAACAGGACAGCAACCG | ATCAGCAAGTGTGAAGCCAGCAG |
| *Aarg14G028100* | GCAAAATGTGGTGGCAGTGGTTG | CAAGACCAGCCTTCAGCACACTC |
| *Aarg16G024750* | ACCAGGTGCCCATCTCTCAGC | AGCAGCCTAAGCATTCGGTCAAG |
| *Aarg17G004080* | GCACCAGTCGCACCACCAATAG | CGTGCTCCTCTTCGGTCTTCTTG |
| *Aarg17G011270* | GCTGCTGCCCTTCCTCTTGC | CCAACTCCACCAGCACCATTCAG |

**Supplementary Table 2.** Sequencing data quality preprocessing results.

| **Sample** | **Raw bases (G)** | **Clean bases (G)** | **Q20 (%)** | **Q30 (%)** | **GC (%)** | **Reads mapped** |
| --- | --- | --- | --- | --- | --- | --- |
| AS_1 | 6.40G | 6.30G | 98.81 | 96.36 | 43.64 | 77.6% |
| AS_2 | 6.38G | 6.29G | 98.80 | 96.3 | 43.36 | 76.21% |
| AS_3 | 6.86G | 6.75G | 98.82 | 96.35 | 43.68 | 75.82% |
| AA_1 | 6.54G | 6.44G | 98.88 | 96.52 | 43.67 | 81.31% |
| AA_2 | 6.07G | 5.96G | 98.81 | 96.35 | 44.00 | 80.61% |
| AA_3 | 6.00G | 5.87G | 98.87 | 96.52 | 44.04 | 81.43% |

**Supplementary Table 3.** Top 30 metabolites with the largest absolute value of Log_2_FC in the AS vs. AA comparison group (HS-SPME-GC-MS).

| **Compounds** | **Class I** | **Log_2_FC** |
| --- | --- | --- |
| Methenamine | Amine | -14.63 |
| Decanal | Aldehyde | -11.39 |
| 4-Undecanone | Ketone | -9.87 |
| 1-Octanol, 3,7-dimethyl- | Alcohol | -9.67 |
| Phenol, 2-nitro- | Phenol | -9.39 |
| Benzoic acid, 3-chloro- | Acid | -9.33 |
| Benzoic acid, 2-chloro- | Acid | -9.33 |
| Benzoic acid, 4-chloro- | Acid | -9.33 |
| (2R,4R)-4-Methyl-2-(2-methylprop-1-en-1-yl) tetrahydro-2H-pyran | Terpenoids | -8.79 |
| 2-Acetyl-1,3-cyclohexanedione | Ketone | -8.46 |
| 6-Octen-1-ol, 7-methyl-3-methylene- | Terpenoids | -8.20 |
| Pyridine, 2-propyl- | Heterocyclic compound | -8.00 |
| 1,2-Octanediol | Alcohol | -7.90 |
| 2-Decanol | Alcohol | -7.87 |
| [1,1'-Bicyclopentyl]-2-one | Ketone | -7.80 |
| Diethanolamine | Amine | -7.75 |
| Benzene, (1-methylpropyl)- | Aromatics | -7.59 |
| Benzene, (2-methylpropyl)- | Aromatics | -7.59 |
| Butanoic acid, 4-hexenyl ester, (Z)- | Ester | -7.58 |
| Naphtho[2,1-b] furan | Heterocyclic compound | -7.54 |
| Isopentyl hexanoate | Ester | -7.31 |
| (2S,4R)-4-Methyl-2-(2-methylprop-1-en-1-yl) tetrahydro-2H-pyran | Terpenoids | -7.25 |
| 2H-Pyran, tetrahydro-4-methyl-2-(2-methyl-1-propenyl)- | Terpenoids | -7.25 |
| trans-Rose oxide | Terpenoids | -7.25 |
| Phenylacetic acid propyl ester | Ester | -7.18 |
| 2-Nonen-4-one | Ketone | -7.18 |
| alpha-Irone | Terpenoids | -7.07 |
| Undecane, 4,8-dimethyl- | Hydrocarbons | -7.07 |
| Undecane, 3,8-dimethyl- | Hydrocarbons | -7.07 |
| Undecane, 3,4-dimethyl- | Hydrocarbons | -7.07 |

**Supplementary Table 4.** Top 30 metabolites with the largest absolute value of Log_2_FC in the AS vs. AA comparison group (UPLC-ESI-MS).

| **Compounds** | **Class Ⅱ** | **Log2FC** |
| --- | --- | --- |
| Chrysoeriol 7-O-Neohesperidoside* | Flavones | -9.88447387 |
| Diosmetin-7-O-Neohesperidoside (Neodiosmin)* | Flavones | -9.61185306 |
| Kaempferide 3-rutinoside* | Flavonols | -9.61185306 |
| 5,3'-Dihydroxy-7,4'-Dimethoxyflavone | Flavones | -8.52781786 |
| 4',6-Dihydroxy-5,7-dimethoxyflavanone | Flavanones | 8.223440942 |
| Wogonin | Flavones | -7.18220125 |
| Pinocembrin | Flavanones | 7.161958756 |
| Acacetin* | Flavones | -6.68244502 |
| Limocitrin (5,7,4'-trihydroxy-8,3'- dimethoxyflavone) * | Flavonols | -6.56613448 |
| 3,3',4',5'-Tetrahydroxy-5,7-Dimethoxyflavone* | Flavonols | -6.56613448 |
| Rivularin* | Flavones | -6.5059031 |
| Eupatilin* | Flavones | -6.5059031 |
| Luteolin-7-O-rutinoside* | Flavones | 6.124334537 |
| Izalpinin* | Flavonols | -6.18921314 |
| 5-Hydroxyauranetin | Flavonols | -6.13445298 |
| 7,8-Dihydroxy-5,6,4'-trimethoxyflavone* | Flavones | -6.1264774 |
| Tenaxin I* | Flavones | -6.1264774 |
| Luteolin-7-O-rutinoside* | Flavones | 6.124334537 |
| Kaempferol-3-O-glucorhamnoside* | Flavonols | 5.636763125 |
| Eupatilin-7-O-glucoside* | Flavones | -5.63008318 |
| Mikanin 3-galactoside* | Flavones | -5.63008318 |
| rhamnazin-3-O-β-D-apiosyl-(1→2)-O-β-D-glucoside | Flavonols | -5.60644642 |
| 3',5-Dihydroxy-4',6,7-trimethoxyflavanone* | Flavanones | 5.567667763 |
| 4'-(beta-D-glucopyranosyloxy)-5-hydroxy-3,3',7-trimethoxyflavone | Flavonols | -5.54266222 |
| Tricin-5-O-(6'-O-malonyl) glucoside | Flavones | -5.43711664 |
| Tricin-7-O-(6''-xylosyl) glucoside* | Flavones | -5.39969994 |
| Tricin-7-O-arabinosyl (1→6)glucoside* | Flavones | -5.39969994 |
| 6,7,8-Tetrahydroxy-5-methoxyflavone* | Flavones | -5.35350614 |
| Kaempferol-3-O-neohesperidoside* | Flavonols | 5.338235213 |
| Scolymoside* | Flavones | 5.338235213 |
